# Critical amino acid residues regulating TRPA1 Zn^2+^ response: A comparative study across species

**DOI:** 10.1016/j.jbc.2024.107302

**Published:** 2024-04-18

**Authors:** Masaki Matsubara, Yukiko Muraki, Hiroka Suzuki, Noriyuki Hatano, Katsuhiko Muraki

**Affiliations:** Laboratory of Cellular Pharmacology, School of Pharmacy, Aichi Gakuin University, Nagoya, Japan

**Keywords:** transient receptor potential channels (TRP channels), zinc, mutagenesis, single-nucleotide polymorphisms (SNP), protein motif, MIB2, metal geometry, AlphaFold database

## Abstract

Cellular zinc ions (Zn^2+^) are crucial for signal transduction in various cell types. The transient receptor potential (TRP) ankyrin 1 (TRPA1) channel, known for its sensitivity to intracellular Zn^2+^ ([Zn^2+^]_i_), has been a subject of limited understanding regarding its molecular mechanism. Here, we used metal ion-affinity prediction, three-dimensional structural modeling, and mutagenesis, utilizing data from the Protein Data Bank and AlphaFold database, to elucidate the [Zn^2+^]_i_ binding domain (IZD) structure composed by specific AAs residues in human (hTRPA1) and chicken TRPA1 (gTRPA1). External Zn^2+^ induced activation in hTRPA1, while not in gTRPA1. Moreover, external Zn^2+^ elevated [Zn^2+^]_i_ specifically in hTRPA1. Notably, both hTRPA1 and gTRPA1 exhibited inherent sensitivity to [Zn^2+^]_i_, as evidenced by their activation upon internal Zn^2+^ application. The critical AAs within IZDs, specifically histidine at 983/984, lysine at 711/717, tyrosine at 714/720, and glutamate at 987/988 in IZD1, and H983/H984, tryptophan at 710/716, E854/E855, and glutamine at 979/980 in IZD2, were identified in hTRPA1/gTRPA1. Furthermore, mutations, such as the substitution of arginine at 919 (R919) to H919, abrogated the response to external Zn^2+^ in hTRPA1. Among single-nucleotide polymorphisms (SNPs) at Y714 and a triple SNP at R919 in hTRPA1, we revealed that the Zn^2+^ responses were attenuated in mutants carrying the Y714 and R919 substitution to asparagine and proline, respectively. Overall, this study unveils the intrinsic sensitivity of hTRPA1 and gTRPA1 to [Zn^2+^]_i_ mediated through IZDs. Furthermore, our findings suggest that specific SNP mutations can alter the responsiveness of hTRPA1 to extracellular and intracellular Zn^2+^.

The transient receptor potential ankyrin 1 (TRPA1) channel, a nonselective cation channel, is widely expressed in nonneuronal as well as neuronal cells in mammals. TRPA1 serves as a biological sensor, responding to environmental irritants and oxidative- and thiol-reactive compounds (1, 2, 3, 4, 5), making it a potential biological sensor against endogenous and exogenous factors. Because transgenic mice lacking TRPA1 exhibit lower sensitivity to mechanical stimulation, cold stimuli, and tumor necrosis factor-α-induced mechanical hyperalgesia (6, 7, 8, 9, 10), TRPA1 has been proposed as a potential nociceptor mediating acute and inflammatory pain (9, 10, 11, 12, 13). Of particular interest are TRPA1's unique properties, such as its ability to import external Zn^2+^ into cells and its sensitivity to nanomolar concentrations of intracellular Zn^2+^ ([Zn^2+^]_i_) for activation, implicating TRPA1 as a potential Zn^2+^ regulator in biological systems (14, 15). A series of permeability and structure-function experiments revealed a mechanism whereby the entry of Zn^2^⁺ through the channel pore facilitates interactions with intracellular cysteine and histidine residues (16, 17). The biological relevance of these findings is highlighted by studies indicating that mice deficient in TRPA1 exhibit reduced nociceptive responses to zinc acetate injections in their hindpaws (17). Furthermore, introducing ZnCl₂ into the respiratory tract significantly decreases the breathing rate in mice, a phenomenon not seen in animals without TRPA1 (18). TRPA1 is also intrinsically activated by endogenous [Zn^2+^]_i_ in inflammatory human knee joint fibroblast-like synoviocytes (19). However, even though TRPA1 is permeable to external Zn^2+^ and subsequent elevation of [Zn^2+^]_i_ can facilitate to activate TRPA1 (14, 17), our understanding of the mechanisms governing Zn^2+^ regulation by TRPA1 remains limited.

To identify critical AAs for the regulation of ion channel function in human, extensive studies have been performed. Single-nucleotide polymorphism (SNP) databases (DBs) have proven valuable in elucidating the roles of specific AAs in channel function, both physiologically and pathophysiologically. However, mutation-based approaches often prove inconclusive and unsuccessful. On the other hand, mutagenic studies in model animals hold promise, allowing for the genetic manipulation of specific AAs in channel proteins to induce gain-of-function or loss-of-function (LOF) phenotypes (6, 7, 8, 9, 10). Nevertheless, establishing a direct link between mutation and phenotype remains unpredictable even in such experiments. Alternatively, comparing channel functions between humans and other species can provide insights into critical AAs in human proteins. Indeed, comparative studies of TRPA1 have revealed significant AAs governing its function, including heat-sensitive AAs (20), menthol-binding AA (21), A-967079 binding AA (22), and AAs involved in external divalent cation interactions (23). Furthermore, by comparing TRPA1 between humans and mice, it has been recently discovered that a potent TRPA1 agonist, JT010, specifically activates human TRPA1 (hTRPA1) but not mouse TRPA1 (mTRPA1) (24, 25, 26).

In the present study, we have identified two sets of four AAs in TRPA1 that constitute [Zn^2+^]_i_ binding domains (IZDs) in both hTRPA1 and chicken TRPA1 (gTRPA1). We used metal ion-affinity (MIA) prediction by metal ion-binding site prediction and modeling server (MIB2, (27, 28, 29)), 3D structural modeling with Metal Geometry in UCSF chimera (30), and mutational analysis, based on data from the Protein Data Bank (PDB) and AlphaFold DB (31, 32). Additionally, among several mutants of extracellular surface site AAs in hTRPA1, a mutation replacing arginine with histidine at position 919 (R919H) alone abolished the response to external Zn^2+^. While MIA prediction and 3D modeling suggested an extracellular Zn^2+^ binding domain-like (EZDL) structure in hTRPA1 with R919H and in gTRPA1, our mutational experiments revealed that external Zn^2+^ does not interact with these EZDLs. When evaluating the Zn^2+^ response in SNP variants located in IZDs and EZDL, we found a significant reduction in Zn^2+^ responses in mutants with SNP-induced substitutions: tyrosine to asparagine at position 714 and arginine to proline at position 919. These findings suggest that such substitutions could lead to an LOF phenotype in hTRPA1.

## Results

**Figure 1:**
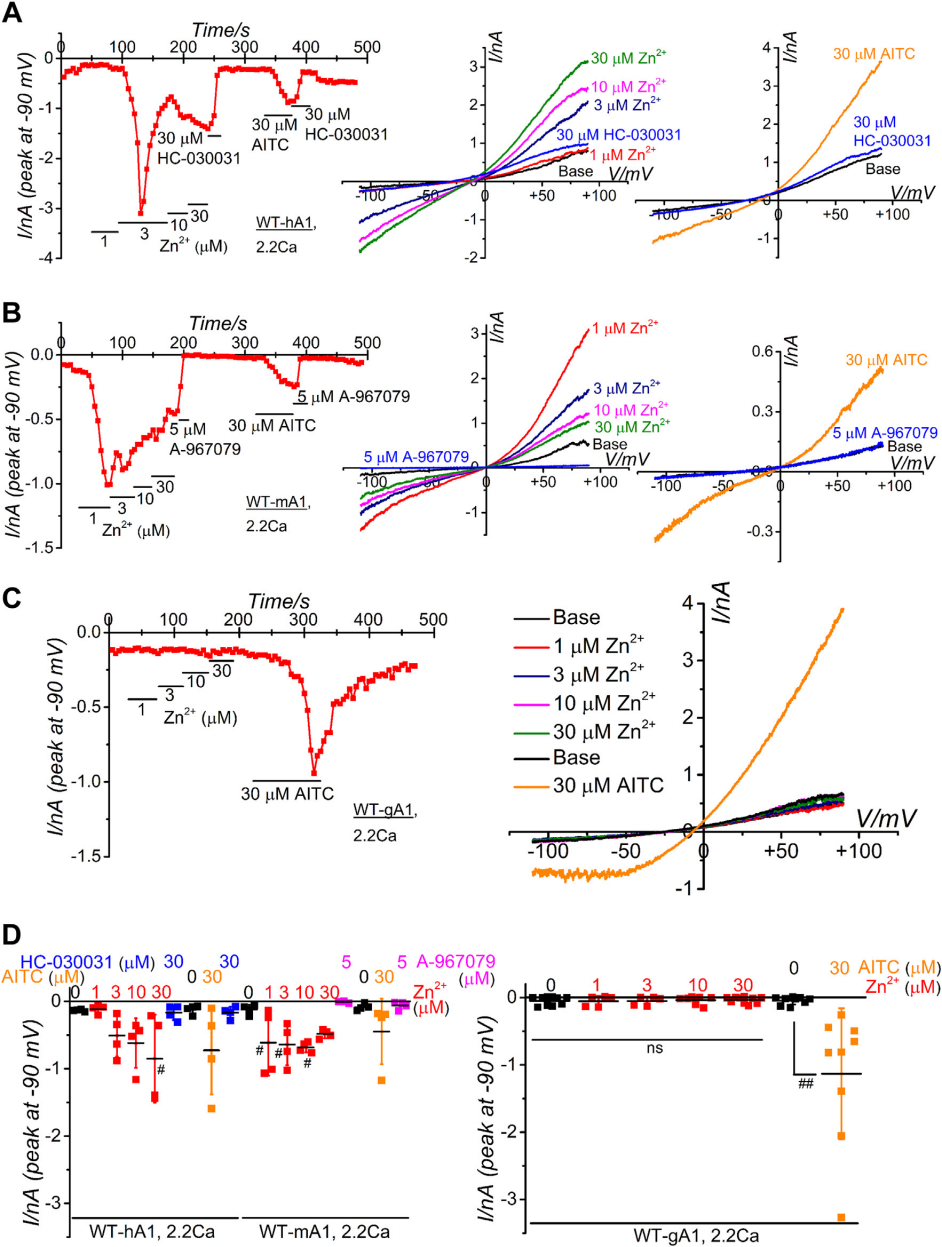
**Comparative analysis of Zn**^**2+**^**-dependent TRPA1 activation across species.***A*–*D*, comparative assessment of Zn^2+^-induced TRPA1 channel currents in representative HEK cells expressing WT-hA1 (*A*), WT-mA1 (*B*), and WT-gA1 (*C*). Cells were superfused with SBS containing 2.2 mM Ca^2+^ and dialyzed with Cs-aspartate rich pipette solution including 0.3 μM Ca^2+^. Ramp waveform voltage pulses ranging from −110 to +90 mV were applied for 300 ms every 5 s at a holding potential of −10 mV. Each cell was exposed to Zn^2+^ to evaluate its effects on membrane currents at −90 mV. Following treatment with the final concentration of Zn^2+^, 30 μM HC-030031 (*A* and *D*) or 5 μM A-967079 (*B* and *D*) was applied. To assess TRPA1 functional expression, AITC (30 μM) was applied. Time-course of peak inward current changes at −90 mV (*left panel*) and current-voltage relationships (I-V) of Zn^2+^- and AITC-induced TRPA1 (*middle* and *right panels*) are shown. *D*, peak amplitudes of inward currents induced by Zn^2+^ and AITC at −90 mV were plotted and averaged for WT-hA1 (four independent experiments; ^#^*p* < 0.05 by Dunnett test), WT-mA1 (four independent experiments; ^#^*p* < 0.05 by Dunnett test), and WT-gA1 (5–10 independent experiments, ^##^*p* < 0.01 by paired-*t* test). AITC, aryl-isothiocyanate; HEK, human embryonic kidney; SBS, standard bathing solution; TRPA1, transient receptor potential ankyrin 1.

To examine the expression of WT and mutant TRPA1 proteins in human embryonic kidney (HEK) cells, their responses to the conventional electrophilic agent, aryl-isothiocyanate (AITC) were assessed at the end of experiments (4). Additionally, the expression of WT and mutant human TRPA1 (hTRPA1) was confirmed at the protein level using four different antibodies (Fig. S1, *A*–*G*). Unfortunately, specific antibodies against mouse TRPA1 (mTRPA1) and chicken TRPA1 (gTRPA1) could not be identified (Fig. S1, *A*–*C*). HEK cells expressing WT hTRPA1 (WT-hA1, Fig. 1*A*) and WT mTRPA1 (WT-mA1, Fig. 1*B*) were utilized to examine the effects of external Zn^2+^ in a concentration range of 1 to 30 μM on WT-hA1 and WT-mA1 in the standard bathing solution (SBS) containing 2.2 mM Ca^2+^. As shown in Figure 1, *A* and *B*, treatment of HEK cells expressing WT-hA1 and WT-mA1 with external Zn^2+^ induced inward currents at −90 mV, which were subsequently inhibited by TRPA1 antagonists (30 μM HC-030031 and 5 μM A-967079 in Fig. 1, *A* and *B*, respectively). However, in SBS with Ca^2+^, the Zn^2+^-induced responses were occasionally transient and exhibited variability from cell to cell in both WT-hA1 and WT-mA1 (left panel of Fig. 1, *A*, *B*, and *D*). In contrast, under identical experimental conditions, external Zn^2+^ failed to induce inward currents in HEK cells expressing WT TRPA1 of chicken (WT-gA1). However, AITC effectively induced TRPA1-like currents (Fig. 1*C* and right panel of Fig. 1*D*), indicating that the response of the TRPA1 channel to external Zn^2+^ varies among species.

**Figure 2:**
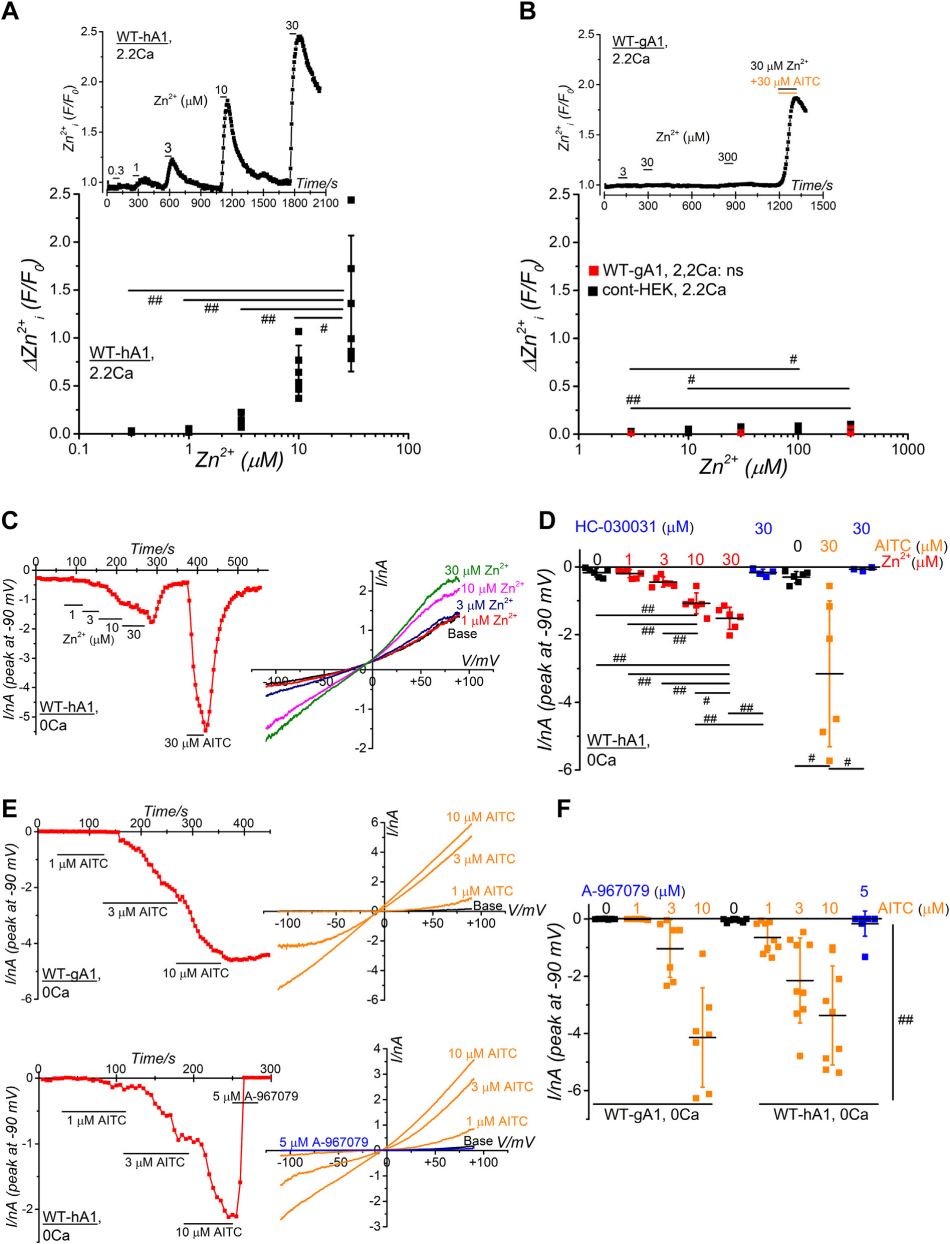
**Cellular [Zn**^**2+**^**]**_**i**_**components and functional comparisons of hTRPA1 and gTRPA1.***A* and *B*, comparison of cellular [Zn^2+^]_i_ levels in HEK cells expressing WT-hA1 and WT-gA1. Zn^2+^ was also applied to control HEK cells without channel expression (cont-HEK). [Zn^2+^]_i_ levels were monitored in SBS using Fluo-Zin3 (upper panels in A and B). Peak changes in [Zn^2+^]_i_ (ΔZn^2+^_i_ (F/F_o_)) were summarized and plotted under each experimental condition (lower panels in *A* and *B*, 5 independent experiments each, ^#^*p* < 0.05, ^##^*p* < 0.01 by Tukey test). *C* and *D*, Zn^2+^-induced WT-hA1 channel currents were recorded in SBS without external Ca^2+^ (0Ca) in a representative cell. Time-course of peak inward current changes at −90 mV (*left panel*) and I-V relationships under each experimental condition (*right panel*) are displayed (*C*). Peak inward currents induced by Zn^2+^ and AITC at −90 mV were summarized (*D*; 3–6 independent experiments; ^##^*p* < 0.01, ^#^*p* < 0.05 by Tukey test). *E* and *F*, comparison of AITC-induced TRPA1 channel currents in a representative HEK cell expressing WT-hA1 (*E*, *lower*) and WT-gA1 (*E*, *upper*). Peak inward currents induced by AITC at −90 mV were summarized (*F*, 7 and 9 independent experiments in WT-hA1 and WT-gA1, respectively; two-way ANOVA: species; F = 0.859, *p* = 0.358, treatments; F = 37.3, ^##^*p* < 0.001, interaction; F = 2.14, *p* = 0.105). The label “ns” denotes no significance. AITC, aryl-isothiocyanate; HEK, human embryonic kidney; hTRPA1, human TRPA1; SBS, standard bathing solution; TRPA1, transient receptor potential ankyrin 1.

To further substantiate the diminished responsiveness of gTRPA1 to external Zn^2+^ in comparison to hTRPA1, we quantified the change in [Zn^2+^]_i_ in HEK cells expressing WT-hA1 and WT-gA1 using the Zn^2+^ indicator, Fluo-Zin3 (Fig. 2, *A* and *B*). External application of Zn^2+^ ranging from 0.3 to 30 μM led to an increase in [Zn^2+^]_i_ in WT-hA1 (Fig. 2*A*) but not in WT-gA1, even in the presence of 300 μM Zn^2+^ (Fig. 2*B*). Control HEK cells (cont-HEK) lacking TRPA1 expression displayed resistance to external Zn^2+^, with limited sensitivity observed at 100 and 300 μM Zn^2+^ (lower panel in Fig. 2*B*). However, simultaneous application of 30 μM Zn^2+^ and 30 μM AITC induced Zn^2+^-dependent fluorescence changes in HEK cells expressing WT-gA1 (upper panel in Fig. 2*B*, ΔZn^2+^_i_ (F/F0): 0.784 ± 0.219, five independent experiments), indicating that Zn^2+^ permeation through gTRPA1 appeared in the presence of AITC, suggesting that the channel pore gate was widened (33). If both the characteristics, “a nonelectrophilic gTRPA1 agonist” and “a potent inhibitor of hTRPA1” refer to A-967079, it is unclear that 30 μM Zn^2+^ slightly activated WT-gA1 in the presence of A-967079 (Fig. S3*A*). To reduce the variable response to Zn^2+^, we used SBS without Ca^2+^ (0Ca). Cumulative application of Zn^2+^ ranging from 1 to 30 μM induced concentration-dependent inward currents in WT-hA1 (Fig. 2, *C* and *D*). In contrast, 30 μM Zn^2+^ failed to activate WT-gA1 in 0Ca (−0.92 ± 12.8 pA *versus* −14.2 ± 23.2 pA at −90 mV in the absence and presence of 30 μM Zn^2+^, three independent experiments), while 30 μM AITC significantly activated the currents (−30.0 ± 27.5 pA *versus* −237.5 ± 68.6 pA at −90 mV in the absence and presence of 30 μM AITC, three independent experiments, *p* < 0.05 by paired *t* test). Meanwhile, exposure to AITC at concentrations between 1 and 10 μM induced inward currents in both WT-hA1 and WT-gA1 (Fig. 2, *E* and *F*, ^##^*p* < 0.001 by two-way ANOVA), implying comparable sensitivity to AITC between hTRPA1 and gTRPA1.

**Figure 3:**
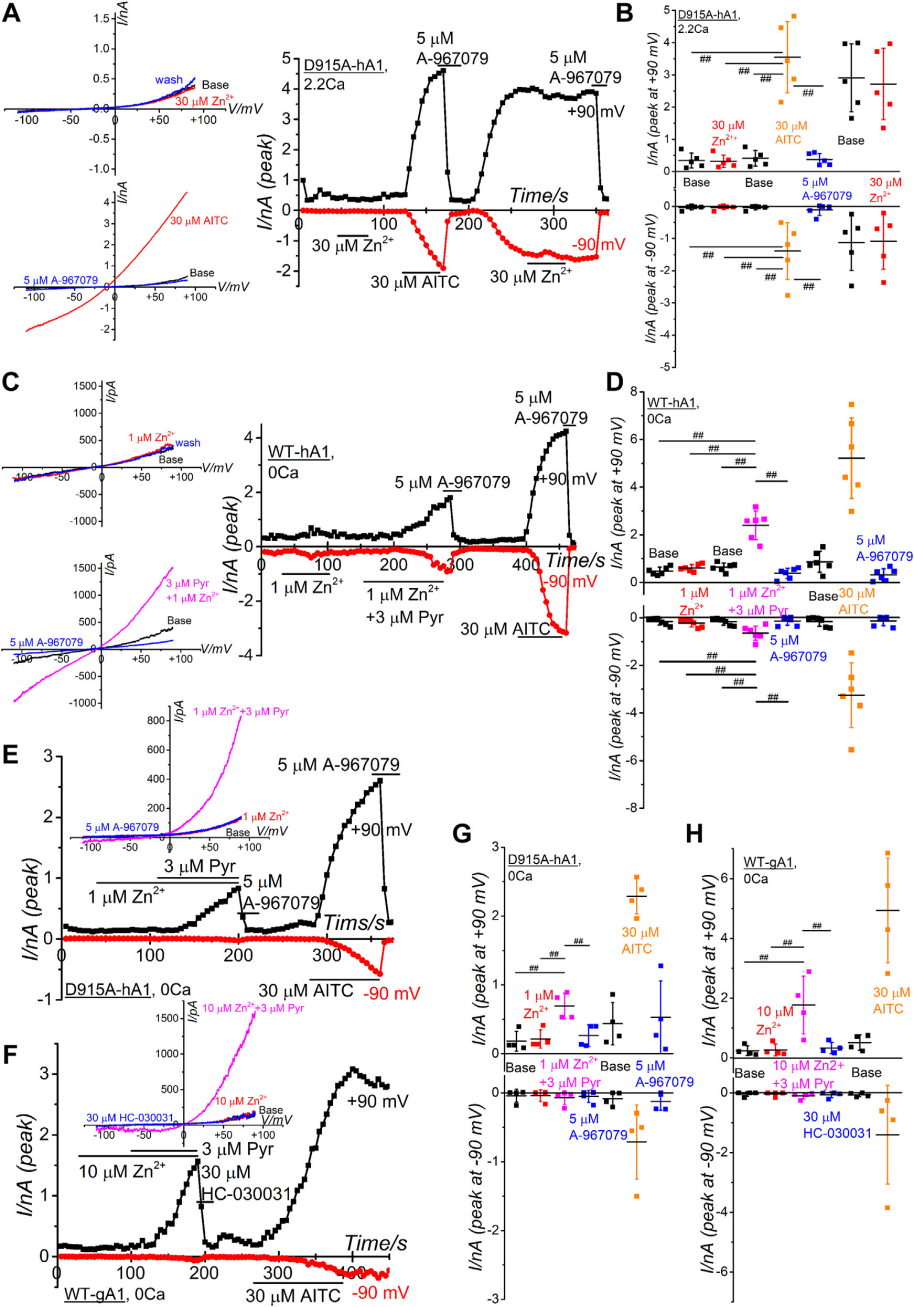
**Functional analysis of TRPA1 mutants at the channel pore upper gate and effects of direct internal application of Zn**^**2+**^**on TRPA1s.***A* and *B*, Zn^2+^-induced TRPA1 channel currents in HEK cells expressing a mutant hTRPA1 (D915A-hA1) with an alanine substitution at residue 915. Representative time-course change (*A*, *right panel*) and I-V relationships (A, *left panels*) of Zn^2+^- and AITC-induced TRPA1 currents in a HEK cell with D915A-hA1 are presented (*A*). Peak currents induced by Zn^2+^ and AITC were plotted at −90 and +90 mV and averaged (*B*; five independent experiments; ^##^*p* < 0.01 by Tukey test). *C*–*H*, direct intracellular application of Zn^2+^ into HEK cells with WT-hA1, D915A-hA1, and WT-gA1. *C*, application of 1 μM Zn^2+^ to WT-hA1 with and without a Zn^2+^ ionophore Pyr. TRPA1 channel activation and expression was confirmed by 5 μM A-967079 and by 30 μM AITC, respectively. I-V relationships under each experimental condition and time-course changes of peak inward and outward currents at −90 and +90 mV are shown in the *left* and *right panels*, respectively. Each peak current amplitude at −90 and +90 mV was plotted and averaged (*D*; six independent experiments; ^##^*p* < 0.01 by Tukey test). *E*–*H*, similar to (*C* and *D*) except application to HEK cells with D915A-hA1 and WT-gA1. I-V relationships under each experimental condition and time-course changes of peak inward and outward currents at −90 and +90 mV are shown in the *upper* and *lower* panel, respectively (*E*). Each peak current amplitude at −90 and +90 mV was plotted and averaged (*G*; four independent experiments; ^#^*p* < 0.05, ^##^*p* < 0.01 by Tukey test). Application of 10 μM Zn^2+^ to WT-gA1 with and without Pyr. TRPA1 channel activation and expression was confirmed by 30 μM HC-030031 and by 30 μM AITC, respectively. I-V relationships under each experimental condition and time-course changes of peak inward and outward currents at −90 and +90 mV are shown in the *upper* and *lower panel*, respectively (*F*). Each peak current amplitude at −90 and +90 mV was plotted and averaged (*H*; four independent experiments; ^#^*p* < 0.05, ^##^*p* < 0.01 by Tukey test). AITC, aryl-isothiocyanate; HEK, human embryonic kidney; hTRPA1, human TRPA1; TRPA1, transient receptor potential ankyrin 1.

Ca^2+^ is one of pivotal endogenous regulators of TRPA1, modulating its activation and inactivation through the influx of Ca^2^⁺ *via* the channel. This dynamic suggests that the Zn^2^⁺-dependent activation of hTRPA1 could be transient in the presence of Ca^2^⁺, as seen when comparing Fig. 1*A* with Fig. 2*C*. This transient nature likely results from an initial amplification of channel activation followed by a rapid cessation of the activity. TRPA1 activation triggered by AITC is also influenced by intracellular Ca^2^⁺ levels in a biphasic manner (34), suggesting that Ca^2^⁺ entering through TRPA1 channels may serve as an intracellular Ca^2^⁺ source for TRPA1 gating regulation. It is anticipated that external Zn^2+^ can also permeate the channel pore of hTRPA1: indeed, the mutation of aspartate (D) at 915 to alanine (A) in hTRPA1 (D915A-hA1; Fig. S1, *F* and *G* for protein expression), which forms the upper channel gate (Fig. 7*A*), abolished the Zn^2+^ response even in the presence of external Ca^2+^, while AITC effectively induced membrane currents in this mutant (Fig. 3, *A* and *B*). Furthermore, Zn^2+^ had little effects on sustained D915A-hA1 currents after the removal of A-967079 (Fig. 3, *A* and *B*), implying that Zn^2^⁺ cannot permeate through the opening gate of D915-hA1. These findings strongly suggest that external Zn^2+^, which is permeable to hTRPA1, cannot pass through D915A-hA1, indicating that WT-gTRPA1 may inherently lack permeability to external Zn^2+^. To investigate whether direct intracellular application of Zn^2+^ can activate D915A-hA1 and WT-gA1, we used a Zn^2+^ ionophore, sodium pyrithione (Pyr) (35). While 1 μM Zn^2+^ alone did not induce activation in WT-hA1, simultaneous addition of 3 μM Pyr led to the activation, which was inhibited by A-967079 (Fig. 3, *C* and *D*). In the presence of 3 μM Pyr, application of 1 μM Zn^2+^ to D915A-hA1 also induced membrane currents sensitive to A-967079 (Fig. 3, *E* and *G*), indicating that Zn^2+^ carried through the ionophore can activate the channel. In contrast, the effect of 1 μM Zn^2+^ in the presence of 3 μM Pyr on WT-gA1 was variable among cells and not statistically significant (Fig. S3, *B* and *C*), whereas 30 μM AITC activated WT-gA1. However, simultaneous application of 10 μM Zn^2+^ and 3 μM Pyr activated WT-gA1, which was inhibited by HC-030031 (Fig. 3, *F* and *H*), strongly suggesting that WT-gA1 is also activated by [Zn^2+^]_i_, similar to WT-hA1.

**Figure 4:**
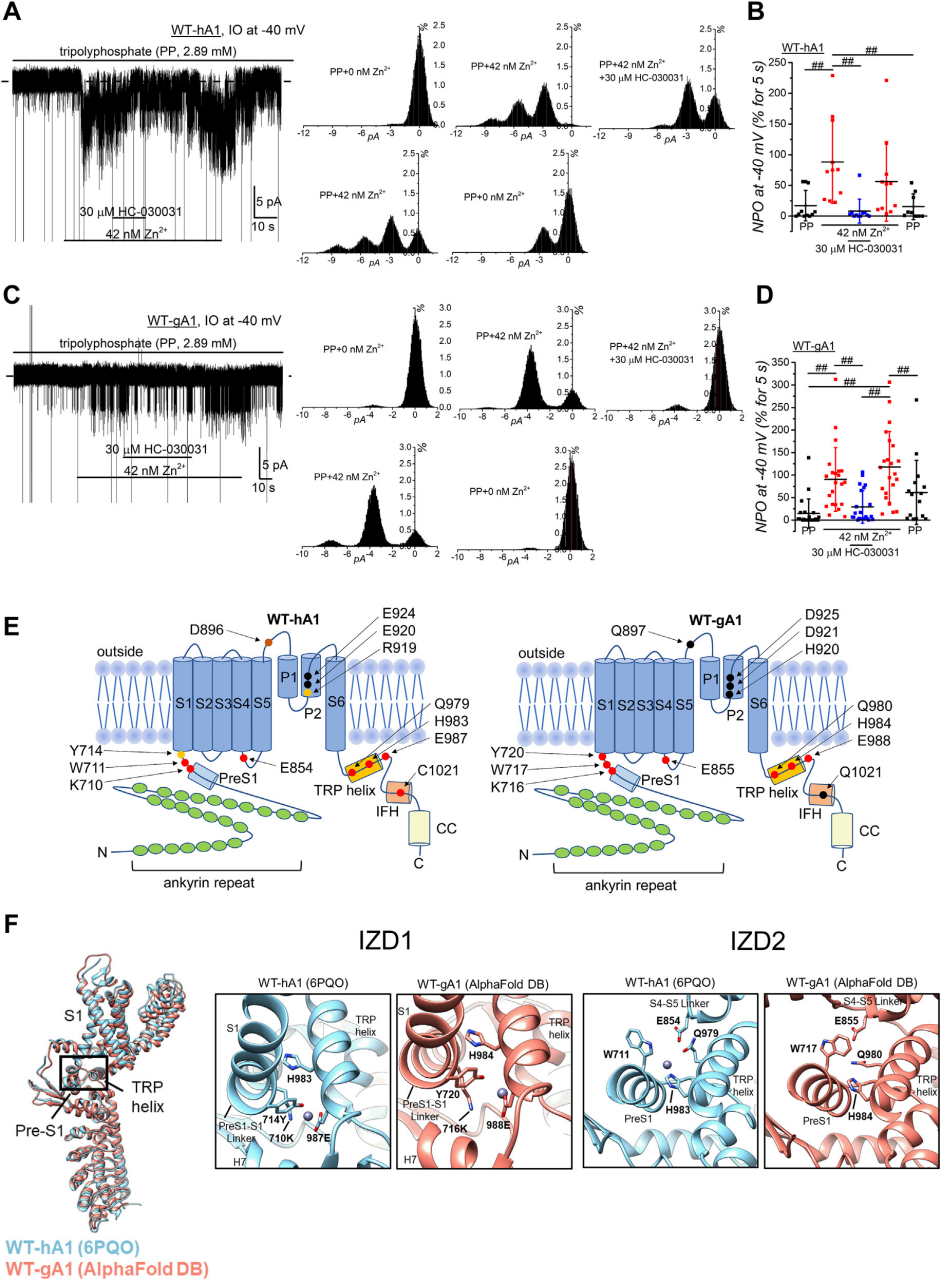
**Intracellular Zn**^**2+**^**induces TRPA1 channel activity in excised inside-out patches and intracellular Zn**^**2+**^**binding model of TRPA1.***A*–*D*, intracellular Zn^2+^ induced TRPA1 channel current at −40 mV in a patch excised from a HEK cell with WT-hA1 (*A* and *B*) and WT-gA1 (*C* and *D*). The bathing solution contained tripolyphosphate (PP) throughout the experiment to maintain the channel activity. The *vertical dashed line* indicates channel close level. In the *right panel*, amplitude histograms for 5 s before and after application of 42 nM Zn^2+^, during addition of 30 μM HC-030031 in the presence of Zn^2+^, after removal of HC-030031 in the presence of Zn^2+^, and after washout of Zn^2+^ from the same patch are shown in WT-hA1 (*A*) and WT-gA1 (*B*). Open probability in multiple channel numbers (NPO) in each experimental condition was plotted and averaged (*B*; 11 independent experiments for WT-hA1 (nine independent experiments for second PP); ^##^*p* < 0.01 by Tukey test, *D*; 22 independent experiments for WT-gA1 (15 independent experiments for second PP); ^##^*p* < 0.01 by Tukey test). *E*, the topological diagrams of WT-hA1 and WT-gA1 include putative Zn^2^⁺-interacting AAs represented by dots, with *red* and *orange dots* distinguishing AAs relevant to Zn^2^⁺ responsiveness. The AAs depicted by *orange* and *brown dots* specifically signify the inclusion of SNP variants analyzed in this study. S, transmembrane segment; P, pore helix; TRP helix, TRP-like helix domain; IFH, interfacial helix; PreS1, Pre S1 helix; CC, coiled coil. *F*, in the *left panel*, the structure model of a single WT-hA1 protomer (6PQO, *blue*, residues from 447 to 1079) was superimposed onto that of WT-gA1 (AF-W8VTH6-F1-model_v4 by AlphaFold DB, *red*, residues from 452 to 1079). The intracellular Zn^2+^ binding domain (IZD) is highlighted in a *boxed* area. In the *middle panel*, using MIB2 docking data (Rank 4 and 13 data for WT-hA1 and WT-gA1, respectively, Table S2), Metal Geometry in UCSF Chimera predicted the composition of IZD1 in WT-hA1 and WT-gA1. For WT-hA1, IZD1 comprises four AAs (H983, E987, K710, and Y714), and for WT-gA1, it includes H984, E988, K716, and Y720. Close-up views of these structures are illustrated. On the *right panel*, IZD2 is reconstructed for both WT-hA1 (comprising H983, Q979, W711, E854, and Rank 88 data (Table S2)) and WT-gA1 (H984, Q980, W717, and E855). The close-up views include Zn^2+^ captured within the domain, except for IZD2 in WT-gA1 (Results and Discussion, and Table S3 in Supporting information). HEK, human embryonic kidney; IZD, [Zn^2+^]_i_ binding domain; SNP, single-nucleotide polymorphism; TRP, transient receptor potential; TRPA1, transient receptor potential ankyrin 1.

**Figure 5:**
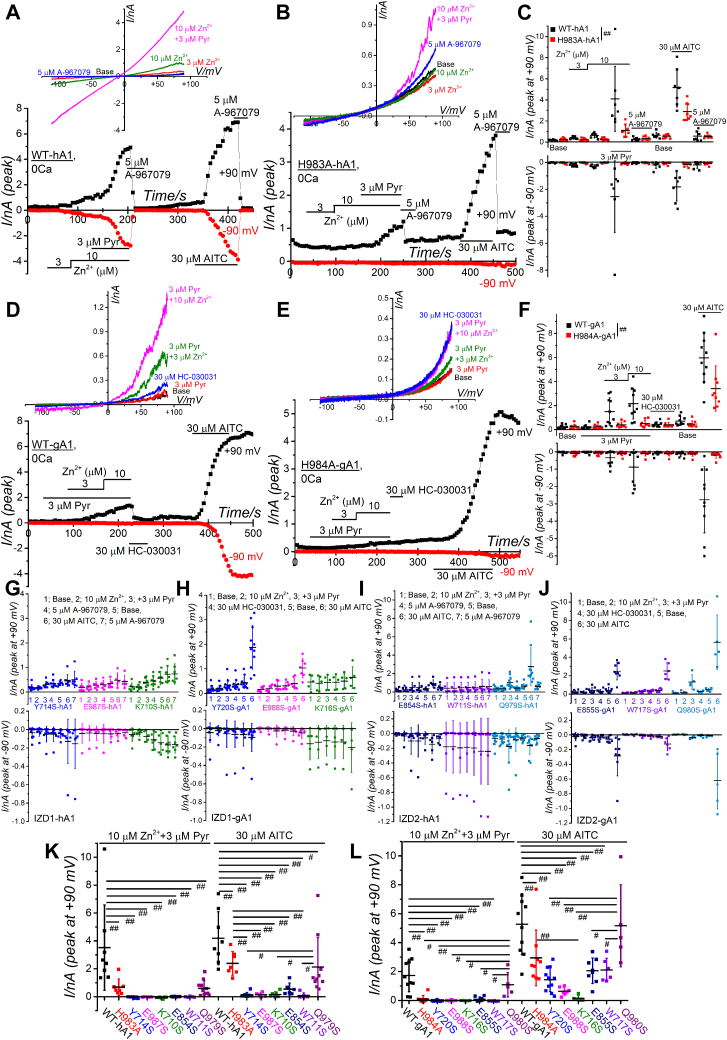
**Mutant-based assay of IZD1 and IZD2 of WT-hA1 and WT-gA1.***A*–*C*, H983 in WT-hA1 was mutated to alanine (H983A-hA1, B), and the Zn^2+^ response was compared with WT-hA1 (*A*). Because hTRPA1 is intrinsically active against external Zn^2+^, 3 and 10 μM Zn^2+^ were applied first, and then 3 μM Pyr was added in the presence of 10 μM Zn^2+^ to induce a direct elevation of intracellular Zn^2+^. The TRPA1 channel activation was confirmed by 5 μM A-967079. TRPA1 function was examined by 30 μM AITC at the end of experiments. I-V relationships under each experimental condition and the time-course changes of peak inward and outward currents at −90 and +90 mV are shown in the *upper* and *lower panels*, respectively (*A* and *B*). Each peak current amplitude at −90 and +90 mV was plotted and averaged (*C*; 7 and 8 independent experiments in WT-hA1 and H983A-hA1, respectively; two-way ANOVA: −90 mV; cell-type; F = 9.29, ^##^*p* = 0.0033, treatments; F = 6.92, ^##^*p* < 0.001, interaction; F = 6.40, ^##^*p* < 0.001, +90 mV; cell-type; F = 8.99, ^##^*p* = 0.004, treatments; F = 16.8, ^##^*p* < 0.001, interaction; F = 6.77, ^##^*p* < 0.001). *D*–*F*, H984 in WT-gA1 was mutated to alanine (H984A-gA1, *E*), and the Zn^2+^ response was compared with WT-gA1 (*D*). Because gTRPA1 is intrinsically inactive against external Zn^2+^, 3 μM Pyr was applied before 3 and 10 μM Zn^2+^. The TRPA1 channel activation was confirmed by 30 μM HC-030031. TRPA1 function was examined by 30 μM AITC at the end of experiments. I-V relationships under each experimental condition and the time-course changes of peak inward and outward currents at −90 and +90 mV are shown in the *upper* and *lower panels*, respectively (*D* and *E*). Each peak current amplitude at −90 and +90 mV was plotted and averaged (*F*; 10 and 9 independent experiments in WT-gA1 and H984A-gA1, respectively; two-way ANOVA: −90 mV; cell-type; F = 5.54, ^#^*p* = 0.021, treatments; F = 4.61, ^##^*p* = 0.0020, interaction; F = 4.31, ^##^*p* = 0.0032, +90 mV; cell-type; F = 21.4, ^##^*p* < 0.001, treatments; F = 12.92, ^##^*p* < 0.001, interaction; F = 8.57, ^##^*p* < 0.001). *G*–*J*, other AAs composing IZD1 and IZD2 in WT-hA1 (IZD1; K710S, Y714S, and E987S, IZD2; W711S, E854S, and Q979S) and WT-gA1 (IZD1; K716S, Y720S, and E988S, IZD2; W717S, E855S, and Q980S) were mutated into serine (S) to test Zn^2+^ response in each mutant. Each peak current amplitude at −90 and +90 mV was plotted and averaged (G; 9, 5–6, and 7 independent experiments in Y714S, E987S, and K710S mutant, respectively, H; 11, 7, and 5 independent experiments in Y720S, E988S, and K716S mutant, respectively, I; 6–7, 6 and 10–11 independent experiments in E854S, W711S, and Q979S mutant, respectively, J; 8, 6, and 5 independent experiments in E855S, W717S, and Q980S mutant, respectively,). *K* and *L*, the delta amplitude at +90 mV before and after application of Zn^2+^ plus Pyr and of AITC in Figure 5, *C*, *F*, *G*, *H*, *I*, and *J* was plotted and averaged in hTRPA1 (*K*) and gTRPA1 (*L*) (^##^*p* < 0.01, ^#^*p* < 0.05 by Tukey test). AITC, aryl-isothiocyanate; hTRPA1, human TRPA1; IZD, [Zn^2+^]_i_ binding domain; TRPA1, transient receptor potential ankyrin 1.

**Figure 6:**
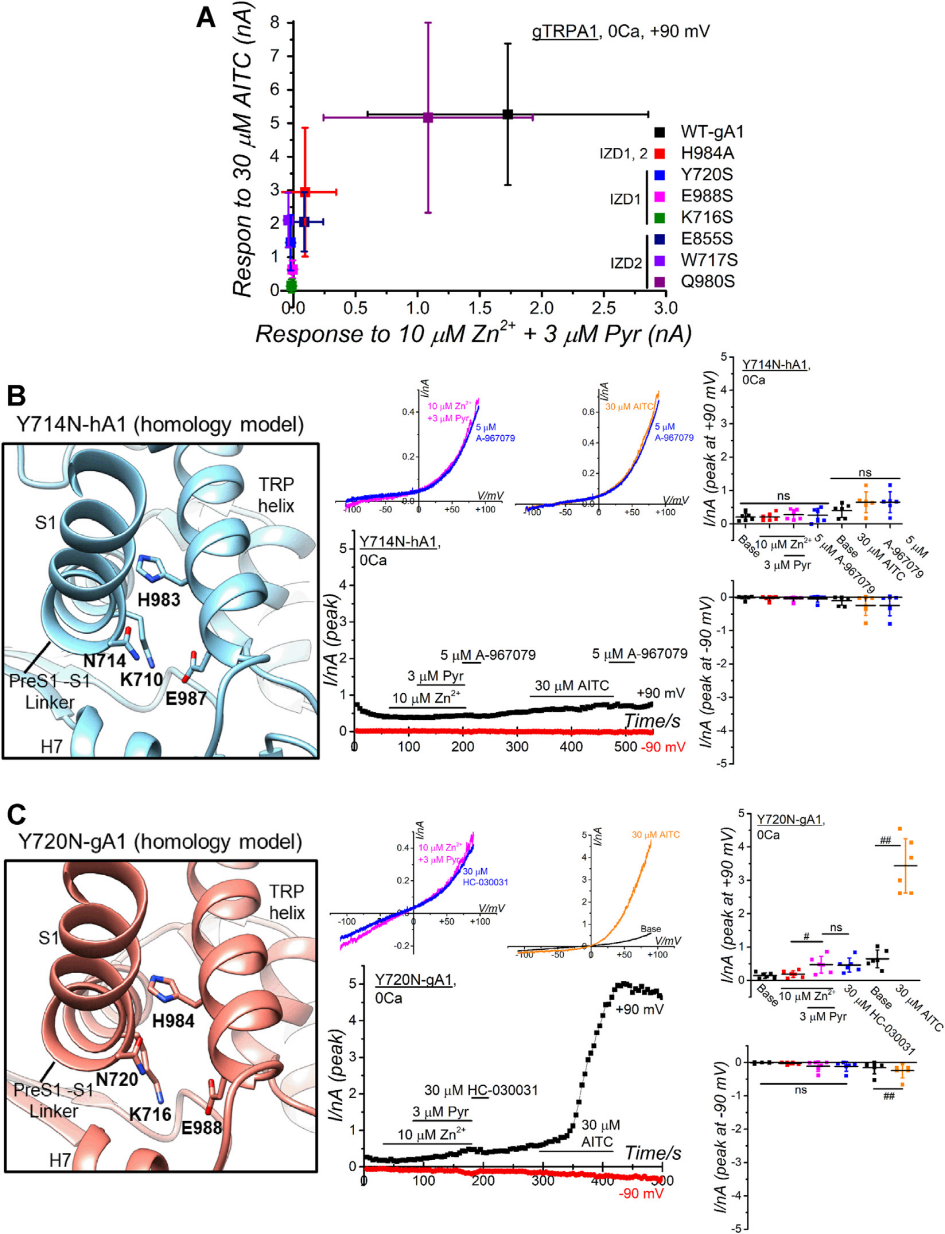
**Mutant-based assay of IZD1 and IZD2 and function of SNP in IZD1.***A*, the delta amplitudes at +90 mV of Zn^2+^ plus Pry-induced currents obtained in Figure 5*L* were plotted against those of AITC-induced currents in IZD1 and IZD2 of gTRPA1. *B* and *C*, the homology model of SNP mutant Y714N-hA1 and the corresponding gTRPA1 mutant with Y720N and the Zn^2+^ response. The homology model of Y714N-hA1 constructed with the model of 6PQO as a template is shown as a close-up view of IZD1 (*B*, *left* panel). The Y720N-gA1 homology model was constructed with the AlphaFold DB model of WT-gA1 as a template. The channel currents induced by 30 μM Zn^2+^ plus Pyr and 30 μM AITC are exhibited in a representative cell in Y714N-hA1 (*B*) and Y720N-gA1 (*C*) (each *middle upper panel*; I-V relationships under each experimental condition, each *middle lower panel*; time-course of peak inward and outward currents at −90 and +90 mV). Each peak current amplitude at −90 and +90 mV was plotted and averaged in the *right panel* (*B*: six independent experiments; ^#^*p* < 0.05 and ^##^*p* < 0.01 by Tukey test; *C*: 6 independent experiments; ^#^*p* < 0.05 and ^##^*p* < 0.01 by Tukey and paired *t* test, respectively). The label “ns” indicates no significance. AITC, aryl-isothiocyanate; DB, database; IZD, [Zn^2+^]_i_ binding domain; SNP, single-nucleotide polymorphism; TRPA1, transient receptor potential ankyrin 1.

**Figure 7:**
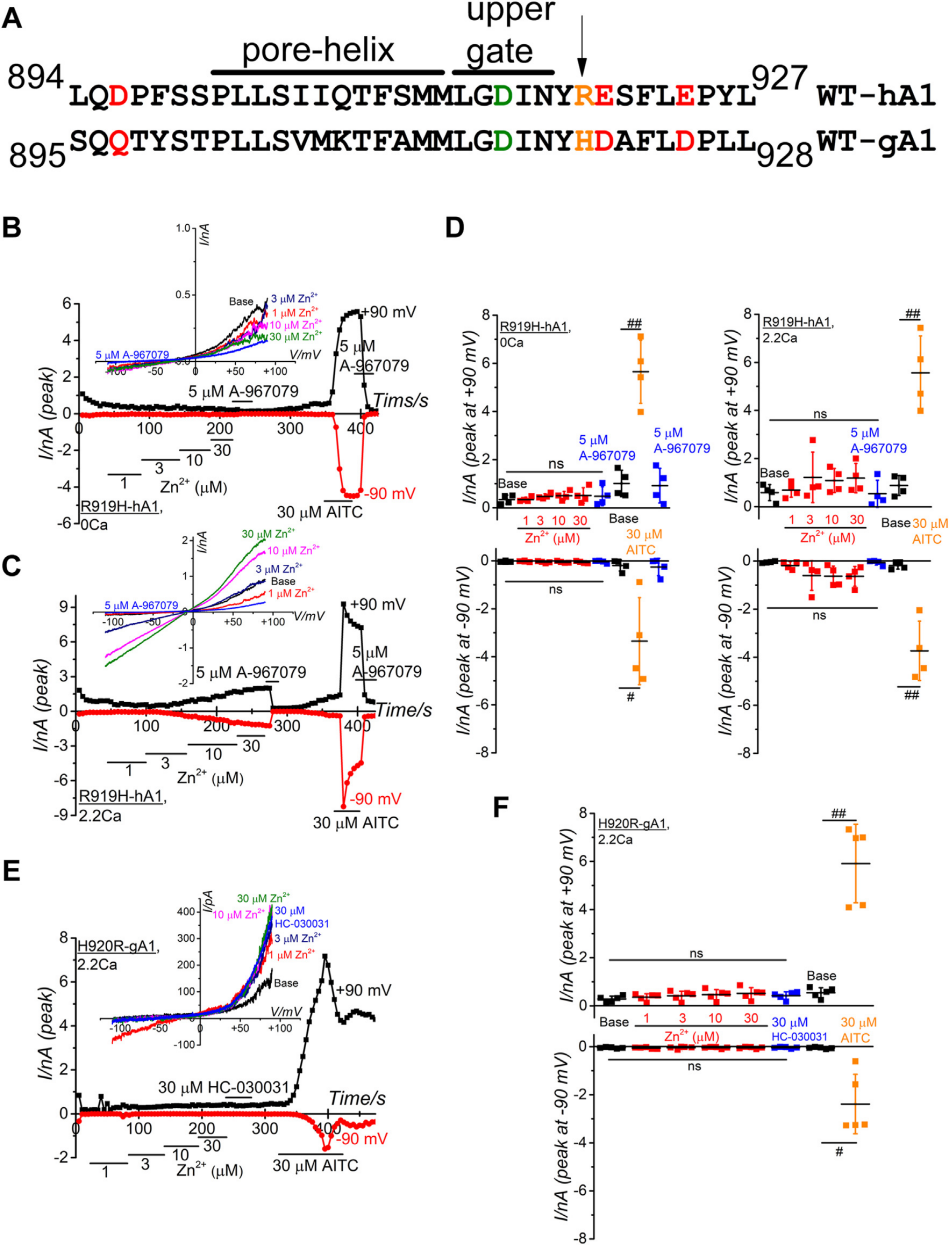
**Critical significance of R919 of hTRPA1 for Zn**^**2+**^**response regulation.***A*, a partial amino acid alignment between WT-hA1 and WT-gA1 revealed substitutions: D896, E920, and E924 in WT-hA1 (highlighted in *red*) were replaced with Q897, D921, and D925 in WT-gA1. D915 in WT-hA1 (in *green*) corresponds to D916 in WT-gA1. An additional substitution (in *orange*) occurs between WT-hA1 (R919) and WT-gA1 (H920) near the exit of the upper channel gate. *B*, R919 of WT-hA1 was mutated into histidine (R919H) to assess its importance. External Zn^2+^ was cumulatively applied to this mutant to test its Zn^2+^ response in the absence (*B* and *D*) and presence of external Ca^2+^ (*C* and *D*). TRPA1 channel activation and expression were confirmed by A-967079 and AITC, respectively. I-V relationships under each experimental condition and time-course changes of peak inward and outward currents at −90 and +90 mV are shown in the *upper* and *lower panel*, respectively. Peak current amplitudes at −90 and +90 mV were plotted and averaged (*D*; four independent experiment each, ^##^*p* < 0.01 by paired *t* test). (*E* and *F*) H920 of gTRPA1 was mutated into H920R. External Zn^2+^ was cumulatively applied to this mutant to test the involvement of H920 in the Zn^2+^ response in the presence of external Ca^2+^. TRPA1 channel activation and expression were confirmed by HC-030031 and AITC, respectively. I-V relationships under each experimental condition and time-course changes of peak inward and outward currents at −90 and +90 mV are shown in the *upper* and *lower* panel, respectively. Peak current amplitudes at −90 and +90 mV were plotted and averaged (*F*; 5 independent experiments, ^##^*p* < 0.01 by paired *t* test). “ns” indicates no significance. AITC, aryl-isothiocyanate; hTRPA1, human TRPA1; TRPA1, transient receptor potential ankyrin 1.

A detailed examination of the structure–function relationship has revealed a mechanism where Zn^2^⁺ entry through the channel pore leads to interactions with intracellular cysteine and histidine residues (17). Additionally, the half-maximal effective concentration for the activation of hTRPA1 in inside-out patch clamp assays is approximately 50 nM (17). To further directly confirm the activation of WT-gA1 by [Zn^2+^]_i_, we applied 42 nM Zn^2+^ to excised inside-out patch membrane isolated from HEK cells expressing WT-hA1 and WT-gA1 (Fig. 4). As previously reported, application of 42 nM [Zn^2+^]_i_ effectively increased channel activity in WT-hA1, which was sensitive to HC-030031 (Fig. 4, *A* and *B*). Similarly, 42 nM [Zn^2+^]_i_ increased the channel activity of WT-gA1, and this response was effectively inhibited by HC-030031 (Fig. 4, *C* and *D*), confirming that gTRPA1 and hTRPA1 are intrinsically sensitive to [Zn^2+^]_i_. The next crucial step is to elucidate the molecular mechanisms underlying the binding of Zn^2+^ to hTRPA1 and gTRPA1. Previous studies have identified cysteine (C1021) and histidine (H983) residues at positions 1021 and 983 of hTRPA1, respectively, as critical for hTRPA1 by [Zn^2+^]_i_ (17). However, the cysteine residue is not conserved in gTRPA1 (Figs. 4*E* and S2, Q1021), leading us to hypothesize that the histidine residue at position 984 (Fig. 4*E*, H984, homologous to H983 in hTRPA1) might play a more significant role in [Zn^2+^]_i_ sensing in gTRPA1. To test this hypothesis and identify any domains involved in [Zn^2+^]_i_ sensing in hTRPA1 and gTRPA1, we modeled potential IZDs using MIA prediction model of Zn^2+^ by the metal ion-binding site prediction and modeling server (MIB2) with 3D cryo-EM data of hTRPA1 at a 2.88 Å resolution (AA residues from 447 to 1079, 6PQO, (24)). This prediction model clearly indicated high affinity scores of histidine at position 983 (H983) and glutamate at position 987 (E987) in WT-hA1 (Tables S1 and S2). Based on MIB2 docking data (Table S2), we utilized Metal Geometry in UCSF Chimera for 3D modeling. This revealed that four AAs—lysine (K) 710, tyrosine (Y) 714, H983, and E987—could potentially coordinate with a single Zn^2+^ ion within a 4 Å distance, forming IZD1 in WT-hA1 (WT-hA1 (6PQO), middle panel of Fig. 4*F*, Results and Discussion, and Table S3 in Supporting Information). Notably, these AAs are conserved in gTRPA1 (K716, Y720, H984, and E988, right panel of Fig. 4*E*). Using AlphaFold DB-predicted 3D structural data (AF-W8VTH6-F1-model_v4) along with MIA prediction model by MIB2 (Tables S1 and S2) and 3D modeling *via* Metal Geometry (Table S3), we inferred an IZD1 in WT-gA1 (middle panel of Fig. 4*F*, WT-gA1 (AlphaFold DB)). Additionally, our analysis suggested that Zn^2+^ could be recognized by another domain, IZD2 (Results and Discussion, and Table S3 in Supporting information), comprising a different set of four AAs—tryptophan (W) 711, E854, H983, and glutamine (Q) 979 in WT-hA1 (Fig. 4*E* and WT-hA1 (6PQO) in right panel of Fig. 4*F*) and their gTRPA1 counterparts, W717, E855, H984, and Q980 (Fig. 4*E* and WT-gA1 (AlphaFold DB) in right panel of Fig. 4*F*). To determine the significance of IZD1 and IZD2 in WT-hA1 and WT-gA1, we next constructed mutants targeting these specific AAs (the protein expression verified by Western blotting (WB) assay in Fig. S1*D*) and tested their responses to [Zn^2+^]_i_.

As previously reported (17), the mutation of H983 to alanine in hTRPA1 (H983A-hA1) significantly reduced the response to [Zn^2+^]_i_-induced currents (Fig. 5, *A*–*C*, and *K*). Similarly, the homologous mutation in gTRPA1, H984A-gA1, dramatically attenuated the [Zn^2+^]_i_-induced current response (Fig. 5, *D*–*F*, and *L*). These results indicate that the histidine residue is crucial for [Zn^2+^]_i_ sensing in both human and chicken TRPA1, with particular significance for gTRPA1, which lacks a cysteine residue at position 1021 (Figs. 4*E* and S2). Additionally, mutations in both IZD1 and IZD2 of hTRPA1 substantially diminished responses to both [Zn^2+^]_i_ and AITC (Fig. 5, *G*, *I*, and *K*). While the protein expression levels of some mutants were lower than that of WT-hA1 (Fig. S1*D*), they were substantially detected, indicating the critical role of these AAs in channel activation of hTRPA1. In gTRPA1, mutations in IZD1 and IZD2, except Q980S, significantly reduced the response to [Zn^2+^]_i_ (Fig. 5, *H*, *J*, and *L*). Notably, all mutants, except K716S, remained sensitive to AITC (Fig. 5, *H*, *J*, and *L*). Comparing the response to Zn^2+^ with the corresponding response to AITC (Fig. 6*A*), all mutants, except K716S, were sensitive to AITC, IZD1 mutants were insensitive to Zn^2+^, and IZD2 mutants with E855S and Q980S remained responsive to Zn^2+^. These findings suggest that IZD1 plays a more critical role in [Zn^2+^]_i_ sensing in TRPA1.

Furthermore, surveying SNP DBs, we identified a SNP at position 714 (original UAC codon, which codes for tyrosine (Y), was mutated into AAC (rs1764318664), encoding asparagine (N)), resulting in a mutant hTRPA1 with Y714N (left panel of Fig. 6*B*, a homology model based on 6PQO, Fig. 4*E* as the topology model). Similar to the mutant with Y714S, neither application of [Zn^2+^]_i_ nor AITC to this SNP mutant (Y714N) elicited membrane current activation (middle and right panels of Fig. 6*B*), with the protein expression detected by WB assay (Fig. S1*D*). This SNP mutant (Y714N) was used to construct the corresponding mutant of gTRPA1 with Y720N (left panel of Fig. 6*C*, a homology model based on AlphaFold DB modeling data). While this mutant channel was activated by AITC, it displayed insensitivity to [Zn^2+^]_i_ (middle and right panels of Fig. 6*C*), indicating that the mutation results in an LOF phenotype against Zn^2+^ in gTRPA1 and an LOF phenotype against Zn^2+^ and AITC in hTRPA1.

It has been shown that negatively charged AAs in the extracellular flanking region of the TRPA1 channel pore are pivotal for Ca^2+^-dependent modulation of heat-induced TRPA1 activation in certain species (23). In gTRPA1, the presence of a noncharged glutamine at position 897 (Q897) is crucial for this regulation, while hTRPA1 substitutes this glutamine with the negatively charged aspartate at position 896 (D896, Fig. 7*A*). Nevertheless, mutation studies suggested that the difference of surface charge does not play an important role in distinct Zn^2+^ response between human and chicken TRPA1 (Fig. S4, *A*–*D*). Meanwhile, we found that an AA residue (arginine at 919 (R919)) near the channel pore exit of hTRPA1 was replaced with histidine (H920) in gTRPA1 (indicated by an arrow in Figs. 7*A* and 4*E*). Moreover, MIA prediction based on AlphaFold DB modeling data clearly indicated high scores for H920, D921, and D925 of gTRPA1 (3.075 each in Table S1, docking data in Table S2). Given histidine's preference for interacting with Zn^2+^, we tested the involvement of H920 in the regulation of Zn^2+^ responses by constructing a mutant of hTRPA1 with R919H (R919H-hA1; Fig. S1, *F* and *G* for protein expression). While 30 μM AITC effectively induced membrane currents in R919H-hA1, external Zn^2+^ up to 30 μM failed to activate this mutant channel (Fig. 7, *B* and *D*). Even in the presence of 2.2 mM Ca^2+^ in SBS (Fig. 7, *C* and *D*), Zn^2+^-induced currents in R919H-hA1 were negligible, underscoring the critical role of this mutation in the response to external Zn^2+^ in hTRPA1. Conversely, we examined the effects of external Zn^2+^ on a mutant gTRPA1, where H920 of gTRPA1 was substituted with arginine (H920R-gA1, Fig. 7, *E* and *F*). Although AITC clearly induced membrane currents, external Zn^2+^ had little effects on this mutant, suggesting that H920 of gTRPA1 is not a sole determinant regulating Zn^2+^ response in gTRPA1.

**Figure 8:**
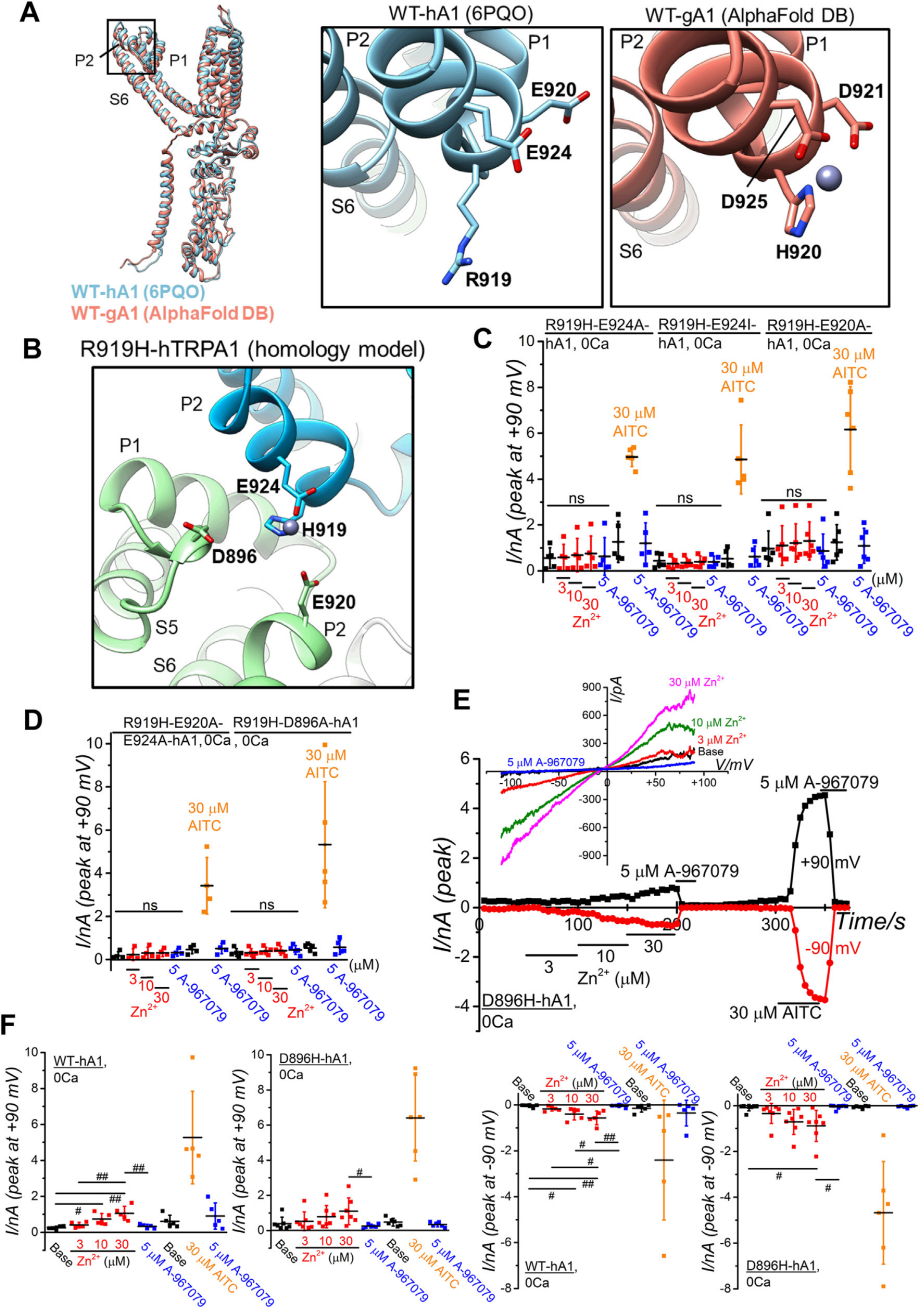
**Mutant-based assay of putative EZDL in WT-hA1 and WT-gA1.***A*, the structure of a single WT-hA1 protomer (6PQO, in *blue*, AAs 447–1079) was superimposed onto a WT-gA1 protomer, modeled based on AlphaFold DB data (AF-W8VTH6-F1-model_v4, in *red*, AAs 452–1079). The putative extracellular Zn^2+^ binding domain-like (EZDL) structure in each protomer is highlighted in a *boxed area*. Close-up views show the potential EZDL in WT-gA1, consisting of residues H920, D921, and D925 (*right panel*) and the corresponding AAs in WT-hA1, including R919, E920, and E924 (*middle* panel). MIB2 suggests a high affinity of these residues in WT-gA1 for Zn^2+^ (Tables S1), and their favorable coordination with Zn^2+^ is predicted by Metal Geometry (Rank 26 data in Tables S2 and S3, Results and Discussion in Supporting information). The Zn^2+^ captured within the EZDL is visible in the WT-gA1 model. *B*, a homology model of the R919H-hA1, potentially forming an EZDL with H919 and E924 on P2 from one subunit (*blue*) and D896 on pre P1 and E920 on P2 from an adjacent subunit (*green*), was constructed using the 6PQO as a template (Results and Discussion in Supporting information). *C* and *D*, to assess the involvement of AAs modeled in EZDL, each residue was mutated in R919H-hA1. Each original AA was mutated to alanine (A) or isoleucine (I). TRPA1 channel activation was confirmed by 5 μM A-967079, and TRPA1 function was examined by 30 μM AITC at the end of experiments. Peak current amplitudes at +90 mV were plotted and averaged (*C*: 5, 5, and 6 independent experiments in R919H-E924A-hA1, R919H-E924I-hA1, and R919H-E920A-hA1, respectively; *D*: 4 and 5 independent experiments in R919H-E920A-E924A-hA1 and R919H-D896A, respectively). *E* and *F*, the channel currents induced by Zn^2+^ and 30 μM AITC are displayed in a representative cell expressing the SNP mutant D896H-hA1 (*E*: *upper panel*; I-V relationships under each experimental condition, *lower panel*; time course of peak inward and outward currents at −90 and +90 mV). *F*, peak current amplitudes of D896H-hA1 at −90 and +90 mV were plotted and averaged (five independent experiments; ^#^*p* < 0.05 and ^##^*p* < 0.01 by Tukey test), and comparisons were made with WT-hA1 (6–7 independent experiments; ^#^*p* < 0.05 and ^##^*p* < 0.01 by Tukey test). AITC, aryl-isothiocyanate; DB, database; SNP, single-nucleotide polymorphism; TRPA1, transient receptor potential ankyrin 1.

We have found the significance of extracellular amino acid residue at position 919 in hTRPA1 for the regulation of Zn^2+^ response (Fig. 4*E* as the topology model). To delve deeper into the molecular mechanisms underlying the importance of R919H, we constructed 3D structural models and designed mutants for investigation. An MIA prediction indicated the presence of EZDL structure composing R919, E920, and E924 in hTRPA1 that could weakly interact with Zn^2+^ (1.807 each for E920 and E924 in Table S1, WT-hA1 (6PQO) in Fig. 8*A*). Moreover, the AAs H920, D921, and D925 in this EZDL of gTRPA1 were hypothesized to be more effective capturing Zn^2+^ due to the presence of histidine (3.075 each for these AAs in Tables S1 and S2) and indeed 3D modeling with Metal Geometry predicted the formation of EZDL in gTRPA1 (WT-gA1 (AlphaFold DB) in Fig. 8*A*, and Table S3). Alternatively, in the R919H mutant, it is possible that Zn^2+^ could be captured by another EZDL constituted of R919H, D896, E920, and/or E924 involving two subunits of hTRPA1 (Fig. 8*B*, and Table S1). Consequently, we generated double and triple mutants of hTRPA1, combining R919H with other mutations with the EZDL (the protein expression verified by WB in Fig. S1*E*). None of these mutants (R919H and E924A; R919H and E924I (glutamate (E) substitution to isoleucine (I) at 924); R919H and E920A in Fig. 8*C*; R919H, E920A, and E924A; R919H and D896A in Fig. 8*D*) exhibited sensitivity to Zn^2+^ while retaining responsiveness to AITC. These suggest that the substitution of the original arginine at position 919 with histidine fundamentally alters the response to external Zn^2+^ in hTRPA1 while playing a minor role in forming a Zn^2+^ binding domain to use these AAs. In our survey of SNP DBs, we identified one SNP at position 896: original GAC codon, translating into aspartate (D), was mutated to CAC (rs145505945), resulting in histidine (H) as the replacement AA. This mutation led to the formation of a mutant hTRPA1 with D896H. While this SNP mutant with D896H exhibited slightly lower sensitivity to external Zn^2+^, the application of 30 μM Zn^2+^ induced substantial membrane currents (Fig. 8, *E* and *F*, the protein expression verified by WB assay in lower panel of Fig. S1*E*), suggesting that this SNP mutation has a minor impact on the response of hTRPA1 to external Zn^2+^.

**Figure 9:**
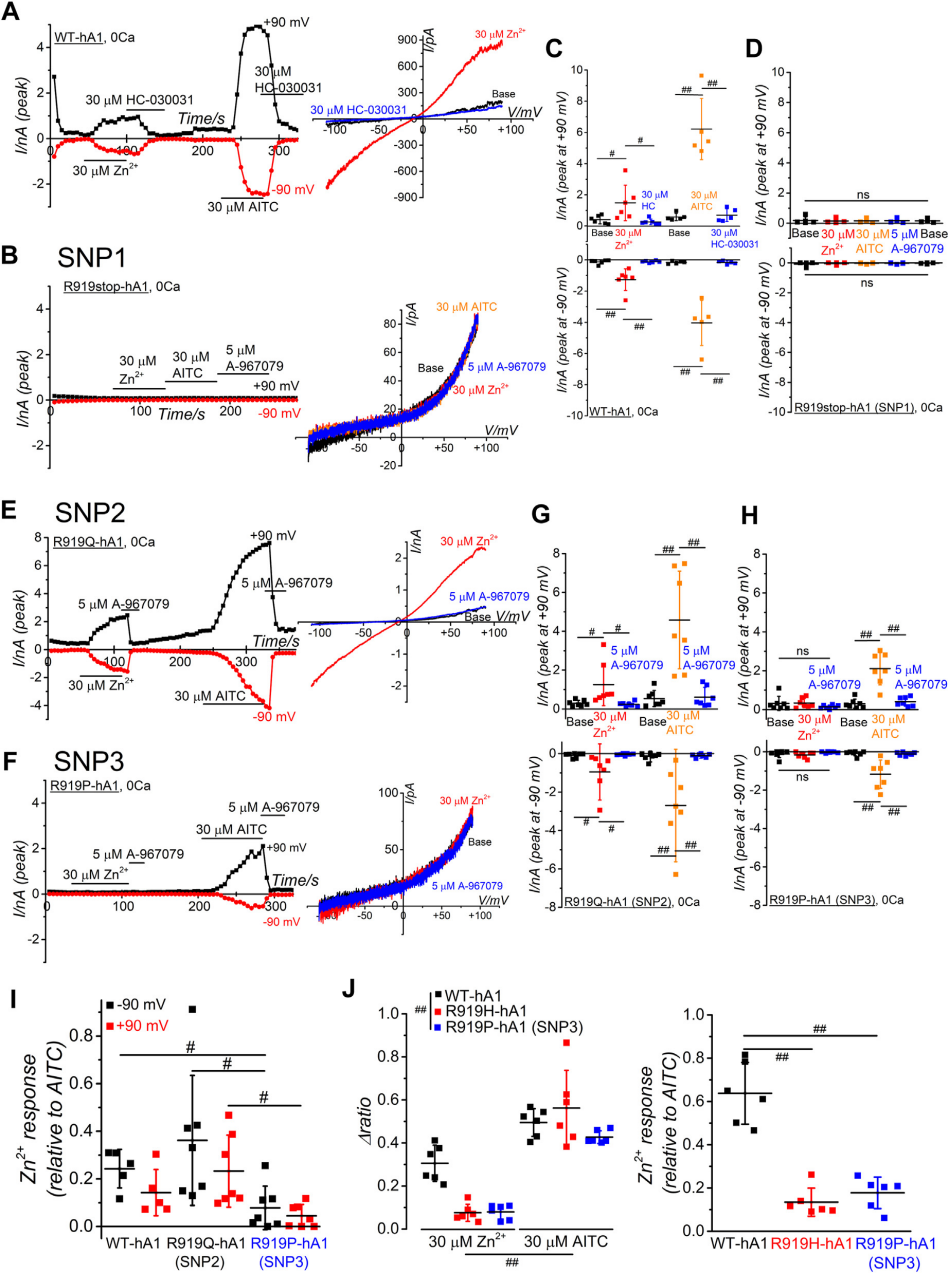
**Zn**^**2+**^**response of WT-hA1 and SNP-induced mutant hTRPA1s at R919.***A*, Zn^2+^-and AITC-dependent activation of membrane currents of WT-hA1. Representative WT-hA1 channel currents induced by 30 μM Zn^2+^ and 30 μM AITC are displayed (*A*, *right panel*; I-V relationships under each experimental condition, *left panel*; time-course of peak inward and outward currents at −90 and +90 mV). *B*–*H*, similar experiments were performed with HEK cells expressing SNP-induced mutants at R919. External Zn^2+^ at 30 μM was applied to each mutant to test Zn^2+^ response. Original WT nucleotides (CGA) coding for R919 of hTRPA1 were mutated into TGA (SNP1), CAA (SNP2), and CCA (SNP3). SNP1 produces a truncated hTRPA1 protein at 919 (*B* and *D*; R919stop-hA1), SNP2 mutates R919 into glutamine (*E* and *G*; R919Q-hA1), and SNP3 mutates R919 into proline (*F* and *H*; R919P-hA1). The TRPA1 channel activation and expression were confirmed by 5 μM A-967079 and 30 μM AITC, respectively. I-V relationships under each experimental condition and time-course changes of peak inward and outward currents at −90 and +90 mV are shown in the *right* and *left panel*, respectively (*B*, *E*, and *F*). *C*, *D*, *G*, and *H*, each peak current amplitude at −90 and +90 mV was plotted and averaged (*C*; 5–6 independent experiments; ^#^*p* < 0.05 and ^##^*p* < 0.01 by Tukey test, *D*; 4–5 independent experiments, *G*; 7 independent experiments; ^#^*p* < 0.05 and ^##^*p* < 0.01 by Tukey test, *H*; 7 independent experiments, ^##^*p* < 0.01 by Tukey test). *I*, peak current amplitude induced by 30 μM Zn^2+^ (Zn^2+^ response) was normalized to that by 30 μM AITC under each experimental condition and plotted (^#^*p* < 0.05 by Tukey test). *J*, effect of Zn^2+^ and AITC on Ca^2+^ response in HEK cells with WT-hA1, mutant R919H-hA1, and SNP3-induced R919P-hA1. The level of [Ca^2+^]_i_ was monitored in SBS and peak change in [Ca^2+^]_i_ (Δratio) was assayed under each experimental condition. Response to 30 μM Zn^2+^ and 30 μM AITC of each HEK cell was plotted and averaged (*left panel*, six independent experiment each, two-way ANOVA: cell-type; F = 8.57, ^##^*p* = 0.0011, treatments; F = 138.0, ^##^*p* < 0.001, interaction; F = 8.70, ^##^*p* = 0.0011). The Δratio induced by 30 μM Zn^2+^ (Zn^2+^ response) was normalized to that by 30 μM AITC under each experimental condition and plotted (^##^*p* < 0.01 by Tukey test). AITC, aryl-isothiocyanate; HEK, human embryonic kidney; hTRPA1, human TRPA1; SBS, standard bathing solution; SNP, single-nucleotide polymorphism; TRPA1, transient receptor potential ankyrin 1.

Furthermore, we explored SNP at position 919 of hTRPA1 in SNP DBs. Three SNP variants were identified at R919 of hTRPA1: original CGA codon, encoding arginine (R), was mutated to TGA (SNP1, rs147706025), resulting in a premature stop codon and generating a truncated hTRPA1 protein at position 919; the CGA codon was altered to CAA (SNP2, rs145505945), leading to a substitution of arginine with glutamine (Q) and producing a mutant hTRPA1 designated as R919Q; the CGA codon was changed to CCA (SNP3, rs145505945), generating proline (P) and leading to a mutant hTRPA1 with R919P (Fig. 4*E* as the topology model). Previous reports (36) have indicated that the truncated hTRPA1 protein due to SNP1 is nonfunctional, as evidenced by its inability to activate membrane currents upon application of AITC (Fig. 9, *A*–*D*). Moreover, this truncated hTRPA1 variant showed no sensitivity to external 30 μM Zn^2+^ (Fig. 9, *A*–*D*), signifying its dysfunctional nature due to the immature protein state (the protein expression verified by WB assay in Fig. S1, *F* and *G*). On the other hand, both Zn^2+^ and AITC induced membrane currents in R919Q-hA1 comparable to those observed in WT-hA1 (Fig. 9, *E* and *G*, the protein expression depicted in Fig. S1, *F* and *G*). On the contrary, akin to R919H-hA1, R919P-hA1 resulting from SNP3, exhibited insensitivity to external Zn^2+^ while maintaining robust responsiveness to AITC (Fig. 8, *F* and *H*). Notably, the AITC response of R919P-hA1 was diminished (Fig. 9*H*), and the protein expression level of R919P-hA1 was marginally lower than other mutants at R919 (Fig. S1, *F* and *G*). Consequently, we reanalyzed the data, normalizing each current amplitude induced by 30 μM Zn^2+^ to that elicited by AITC (Fig. 9*I*). Even after normalization by AITC response, Zn^2+^-induced currents remained smaller in R919P-hA1, indicating that reduced responsiveness of the SNP3-induced mutant to external Zn^2+^. Consistent with this finding, the Ca^2+^ response to external Zn^2+^ was significantly reduced in both R919P-hA1 and R919H-hA1 mutants than WT-hA1 (left panel in Fig. 9*J*). This reduction was evident even when their responses to Zn^2+^ were normalized against those to AITC (right panel of Fig. 9*J*).

## Discussion

In our current investigation, we have successfully identified the presence of two distinct sets of four AAs within TRPA1, referred to as [Zn^2+^]_i_ binding domains, IZD1 and IZD2, in both hTRPA1 and gTRPA1. This discovery was achieved through a combination of MIA prediction modeling of Zn^2+^ binding (MIB2), 3D structural modeling with Metal Geometry, and the generation of specific mutants targeting IZDs, utilizing data from the PDB and AlphaFold DB. Furthermore, amidst various mutation introduced into the extracellular surface AAs of hTRPA1, singular substitution at residue R919 to histidine (R919H) resulted in the abolishment of the response to external Zn^2+^. Although the EZDL of hTRPA1 with R919H and gTRPA1 was proposed through MIA prediction model and 3D simulations, our mutational experiments showed that external Zn^2+^ failed to interact with this EZDL. Among SNP at Y714 in IZD1 and D896 in EZDL, and a triple SNP at R919 in hTRPA1, we found that mutants carrying the Y714N substitution and the R919 substitution to proline exhibited attenuated responses to Zn^2+^. Overall, this study reveals the intrinsic [Zn^2+^]_i_ sensitivity of hTRPA1 and gTRPA1 mediated by IZDs and suggests that certain SNP mutations can modify response of hTRPA1 to both extracellular and intracellular Zn^2+^.

[Zn^2+^]_i_ serves as a pivotal regulator in cellular signal transduction across various cell types. Consequently, TRPA1, known for its permeability to external Zn^2+^ and activation by internal Zn^2+^, plays a significant role as a regulatory protein in Zn^2+^ response in human and mouse cells (14, 17, 19). Our findings indicate that gTRPA1 exhibits a diminished permeability to external Zn^2+^ compared to hTRPA1. Interestingly, in the presence of TRPA1 agonists, the application of 30 μM Zn^2+^ led to elevated [Zn^2+^]_i_ levels (plus 30 μM AITC, Fig. 2*B*) and slightly increased membrane currents susceptible to HC-030031 (plus 5 μM A-967079, Fig. S3*A*), implying that the activation of gTRPA1 by agonists could potentially widen the upper and/or lower gates of the channel. In the presence of TRPA1 agonists, relatively larger cations can permeate hTRPA1 (33). However, the structural data analysis did not reveal distinct differences in pore radii between the mutation-induced channel closed state and the agonist-induced channel open state of hTRPA1 (24). These results emphasize the intricate nature of TRPA1 channel gating and highlight the need for further comprehensive investigations to elucidate the underlying mechanisms governing Zn^2+^ sensitivity in TRPA1 channels across different species.

While gTRPA1 demonstrates low permeability to external Zn^2+^, it shares a similar intrinsic sensitivity to [Zn^2+^]_i_ with hTRPA1. Both gTRPA1 and the Zn^2+^-impermeable mutant D915A-hA1 were efficiently activated by internal Zn^2+^ application, facilitated by the Zn^2+^ ionophore Pyr in a whole-cell clamp configuration. Direct application of 42 nM Zn^2+^ to excised inside-out patch membranes induced HC-030031-sensitive channel activity in both gTRPA1 and hTRPA1, indicating that nanomolar concentrations of [Zn^2+^]_i_ effectively activate gTRPA1 in a manner similar to hTRPA1 (14, 17). In hTRPA1, a mutation of cysteine at position 1021 (C1021) to serine reduced the EC_50_ of external Zn^2+^ response from 1.7 to 5.8 μM, with the maximum response halved compared to WT-hA1 (17). Given that gTRPA1 lacks this critical cysteine residue at 1021 (Figs. 4*E* and S2) for Zn^2+^ response in hTRPA1 (17), we focused on another Zn^2+^-sensitive AA, H983 (hTRPA1) and its homologous H984 in gTRPA1, and finally identified two distinct sets of four AAs in TRPA1 composing IZDs (IZD1: K710, Y714, H983, and E987 in hTRPA1, K716, Y720, H984, and E988 in gTRPA1; IZD2: W711, E854, Q979, and H983 in hTRPA1, W717, E855, Q980, and H984 in gTRPA1). Among these AAs, while MIA modeling by MIB2 predicted high binding scores for H983 (4.294, Table S1) and E987 (4.294, Table S1), the scores for AAs of IZD2 in hTRPA1 were relatively lower, except for H983 (Table S1). The sensitivity of Zn^2+^ binding prediction by MIB2 improved to be 77.1% than MIB (71.1%), yet remained lower than its accuracy for Fe^2+^ (92.5%) and Fe^3+^ (91.6%) (28). Although these seven AAs are conserved in gTRPA1, Zn^2+^ binding scores for IZD1 and IZD2 in gTRPA1 were relatively lower than in hTRPA1 (Table S1). Yet, two AAs in IZD1 of gTRPA1, H984 and E988, were predicted to interact with Zn^2+^ (Table S2), and 3D modelling by Metal Geometry suggested that K716, Y720, H984, and E988 could form IZD1 in gTRPA1 (Fig. 4*F*, and Table S3). In contrast, most mutants in hTRPA1 showed abolished or diminished response to AITC (Fig. 5*K*), implying that mutations in IZDs affect channel activation. AAs K710, Y714, and E987 in IZD1, and W711 and E854 in IZD2 are crucial for hTRPA1 channel activation (37), suggesting that dysfunction in hTRPA1 mutants targeting these AAs could result in diminished Zn^2+^ response. However, our comparative analysis with gTRPA1 disproved this, as substantial AITC responses remained in these mutants, except for K716S (Figs. 5*L* and 6*A*). These findings strongly suggest that IZD1 and IZD2 play crucial roles in Zn^2+^ sensing in TRPA1 and could be potential targets for novel drugs targeting this channel.

We further identified the crucial role of the substitution at R919 of hTRPA1 in response to external Zn^2+^. Specifically, the mutation of R919 in hTRPA1 to histidine, homologous to H920 in gTRPA1, significantly diminished the sensitivity to external Zn^2+^. In gTRPA1, MIA scores of three AAs (H920, D922, and D925) were high (3.075 each in Table S1, 3.075 for the docking sore in Table S2), and indeed Metal Geometry predicted that these AAs are potential components of EZDL (Fig. 8*A*, and Table S3). Therefore, in the R919H-hA1 mutant, it is expected that a set of three or four AAs (D896, H919, E920, and/or E924) composes a putative EZDL (Fig. 8*B*) and indeed, MIB2 (Table S1) predicted high Zn^2+^ binding scores for H919 (4.721), D896 (2.017), E920 (2.894), and E924 (4.721). However, these AAs were not found to be involved in forming the EZDL: double or triple mutants of R919H with D896A, E921A, E924A, or E924I in EZDL in hTRPA1 remained insensitive to external Zn^2+^ (Fig. 8, *C*–*F*), and a mutant with H920R in gTRPA1 showed no response to external Zn^2+^ (Fig. 7, *E* and *F*). It is hypothesized that R919 forms interactions with E920 and S921 in the neighboring protomer (24), and the R919H mutation might alter these interactions, thereby reducing the response to external Zn^2+^. In certain species, negatively charged AAs in the extracellular region of the TRPA1 channel pore play a critical role in the Ca^2+^-dependent modulation of heat-induced TRPA1 activation (23). In fact, noncharged glutamine at position 897 (Q897) in gTRPA1 was replaced with negatively charged aspartate (D896) in hTRPA1. However, it was confirmed that the surface charge of TRPA1 does not influence the response to external Zn^2+^ (Fig. S4, *A*–*D*). When negatively charged aspartate was mutated into histidine at 918 of rat TRPA1 (rTRPA1, D918 homologous to D915 of hTRPA1), the channel activation by external Zn^2+^ was converted to the inhibition (38). In addition, this aspartate residue regulates the permeation of external Ca^2+^ through TRPA1 (39). Recent functional and structural studies of TRPA1 (40) provide valuable insights. The side chains of all four H918 residues in the tetrameric rTRPA1 were found to create a high-affinity binding site for Zn^2+^ (41). Since Zn^2+^ preferentially interacts with the imidazole side chain of histidine, it is plausible that the four H919 residues of the hTRPA1 tetramer might form a similar high-affinity binding site for Zn^2+^. However, the specific positioning of the H919 side chains in hTRPA1 does not align closely with the preferred tetrahedral Zn^2+^ coordination geometry. The specific mechanism through which the R919H mutation attenuates the response to external Zn^2+^ remains unclear.

Upon testing the Zn^2+^ response of the Y714N-hA1 SNP mutant, we observed that the mutant exhibited insensitivity to [Zn^2+^]_i_. Additionally, the application of AITC to this SNP mutant failed to induce any membrane currents. The protein expression of the Y714N SNP mutant, while clearly reduced, was still substantial (Fig. S1*D*), indicating this SNP mutant results in an LOF phenotype. Intriguingly, the homologous mutation, Y720N-gA1, which was responsive to AITC, was insensitive to [Zn^2+^]_i_ (Fig. 6*C*), suggesting that the Y714N SNP mutant lacks channel function independently of deficient IZD1. Among the SNP mutants at R919, the R919P SNP mutant might be an LOF phenotype against external Zn^2+^ due to following reasons: the protein expression was substantial; Zn^2+^ response normalized by AITC response was smaller; Zn^2+^-induced Ca^2+^ response in this SNP mutant was diminished. The mechanisms for the reduction in Zn^2+^ response in R919P-hTRPA1 remain unclear. Proline, being a unique amino acid, might induce structural alterations in the upper channel gate filter, thereby reducing Zn^2+^ permeation. Further structural studies will be essential for comprehending the significance of the critical proline residue at position 919 of hTRPA1. In contrast, a mutant with R919stop, generating truncated hTRPA1 protein, may be dysfunctional, while the heterozygous expression of this mutant with WT-hA1 causes hyperactivity of TRPA1 (36).

Comparative analysis of ion channels across different species is imperative at genetic, protein, and functional levels (42). Previous study has found critical AAs in hTRPA1 that serve as binding sites for A-967079, enabling the comparison of TRPA1 functions between humans and chickens (22). Intriguingly, some of these AAs have been found to be replaced in gTRPA1, transforming A-967079 into an agonist for gTRPA1. Additionally, proton-induced activation of TRPA1 has been demonstrated in humans but not in mice and monkeys, due to specific AAs located within transmembrane domains 5 and 6 (43). Our comparative investigation between hTRPA1 and gTRPA1 has highlighted the significance of IZDs and the amino acid R919 in hTRPA1 for responding to both internal and external Zn^2+^. Furthermore, our survey of SNP DBs has unveiled LOF mutants at positions Y714 and R919 in hTRPA1, where tyrosine and arginine were mutated to asparagine and proline, respectively. A comprehensive comparison across various species promises to yield invaluable insights into the functional aspects of hTRPA1.

## Experimental procedures

### Cell culture

HEK cells obtained from the Health Science Research Resources Bank were cultured in Dulbecco’s modified minimum essential medium (Sigma-Aldrich) supplemented with 10% heat-inactivated fetal calf serum (Sigma-Aldrich), penicillin G (100 U/ml, Meiji Seika Pharma Co, Ltd), and streptomycin (100 μg/ml, Meiji Seika Pharma Co, Ltd).

### Recombinant expression of WT and mutant TRPA1 in HEK cells

HEK cells, at a confluency of 40 to 60%, were transiently transfected with the pcDNA3.1 (Thermo Fisher Scientific) and pIRES2-AcGFP1 (Takara Bio INC) plasmids encoding TRPA1 from human (NM_007332.3), mouse (NM_001348288.1), and chicken (NM_001318460.2) origins. The transfection was performed using lipofectamine 3000 (Thermo Fisher Scientific). Mutant TRPA1s were generated by PCR using mutant oligonucleotide primers. The DNA fragment containing the entire coding region of gTRPA1 was constructed to use complementary DNA derived from the whole brain of chicken (*Gallus gallus* domesticus (GSP (Fayoumi breed)), provided by Avian Bioscience Research Center at Nagoya University through the National Bio-Resource Project of the MEXT, Japan) as a template. For the amplification by RT-PCR, two sets of oligonucleotide primers were designed (5′- CTAGCTAGCGCCGCCACCATGAAGCGCTCTCTGTGG-3′ and 5′- CTGGTTTTGGACAACTGC-3′; 5′- CCGCTCGAGCTACAATAAGCTGCTGCTCTTTTC-3′ and 5′- CCTGTTAAGCGCCAGGGGAC-3′). All constructs were sequenced, and experiments were conducted within 48 h after transfection.

### Western blotting

HEK cells, both with and without WT TRPA1 and those expressing mutant TRPA1s, were lysed in 50 μl lysis buffer composed of 50 mM Tris–HCl 50 (pH 8.0), 150 mM NaCl, 5 mM EDTA, 1% NP-40, 0.5% sodium deoxycholate, 0.1% SDS, supplemented with protease inhibitors. The mixtures were then subjected to a 30-min incubation on ice, with intermittent vortexing at 5-min intervals, followed by centrifugation at 20,000*g* for 30 min at 4 °C. Each lysate, containing 10 μg of protein, was resolved on an 8% polyacrylamide gel. The separated proteins were electrotransferred to a polyvinylidene difluoride membrane. Nonspecific binding of antibodies was blocked by incubation for 2 h in Tris-buffered saline with 5% skim milk and 0.1% Tween-20. For primary antibody incubation, the polyvinylidene difluoride membrane was treated overnight at 4 °C with antibodies specifically targeting C terminus and N terminus of TRPA1 (sc-32353, Lot: H0309, Santa Cruz Biotechnology Inc, Heidelberg Germany; NB110-40763, Lot: NOV-1407-63, NOVUS Biologicals, both at 1:1000 dilution) and other anti-TRPA1 antibodies (ACC-037. Lot: ACC037AN2125, Alomone Lab, KM120, Lot: TG230421, Trans Genic Inc, both at 1:1000 dilution). The membrane was washed thrice with a washing buffer (Tris-buffered saline containing 0.1% Tween-20) and then proved with a secondary antibody (immunoglobulin G-horseradish peroxidase, 1:5000 dilution). After subsequent washes, the detection reagents (Millipore Japan) were incubated, inducing a chemiluminescence signal. To normalize TRPA1 expression, membranes were also treated with a human anti-β-actin monoclonal antibody (A5441, Sigma-Aldrich, at 1:5000 dilution). The resulting blots were visualized using the LAS-4000 illuminator (Fujifilm).

### Patch clamp experiments

Whole-cell and single-channel current recordings were performed as previously described (44). A standard Hepes-buffered bathing solution (SBS) with the following composition (in mM): NaCl 137, KCl 5.9, CaCl_2_ 2.2, MgCl_2_ 1.2, glucose 14, and Hepes 10 [adjusted to pH 7.4 with NaOH]) was used as the extracellular solution. In inside-out patch configuration, the bathing solution contained (in mM): CsCl 140, MgCl_2_ 1, EGTA 1, Hepes 10, tripolyphosphate (PP) 2.89 (pH adjusted to 7.2 with CsOH). For Zn^2+^-buffered external solutions, total Zn^2+^ concentration was determined using the WEBMAXC Standard, following program instructions (14). The resistance of electrodes was 3 to 5 MΩ when filled with the pipette solution. In whole-cell recording, the Cs^+^-rich pipette solution contained (in mM): Cs-aspartate 110, CsCl 30, MgCl_2_ 1, Hepes 10, EGTA 10, and Na_2_ATP 2 (pH adjusted to 7.2 with CsOH). In the inside-out patch configuration, the SBS was used as the pipette solution. To maintain the basal activity of TRPA1 currents under the whole-cell recording conditions, intracellular free Ca^2+^ concentration was maintained at a pCa of 6.5 (0.3 μM Ca^2+^) by adding CaCl_2_ to the pipette solution. Membrane currents and voltage signals were digitized using an analog-digital converter at a 10 kHz sampling rate with a 5 kHz filtering (PCI-6229, National Instruments Japan). Data acquisition and analysis of whole-cell currents and excised inside-out patch currents were performed using WinWCP4.5 and WinEDR3.38, respectively, software developed by Dr John Dempster (University of Strathclyde, UK, https://spider.science.strath.ac.uk/sipbs/software_ses.htm). The liquid junction potential (−10 mV) between the pipette and bath solutions was corrected in whole-cell mode. A ramp voltage protocol from −110 to +90 mV for 300 ms was applied every 5 s from a holding potential of −10 mV. The leak current component was not subtracted from the recorded currents. All experiments were performed at 25 ± 1 °C.

### Fluorescence-based measurement of Zn^2+^ and Ca^2+^

The change in [Zn^2+^]_i_ was measured using FluoZin-3 (35). Cells were incubated with 10 μM FluoZin-3AM for 30-min in SBS at room temperature. Upon excitation at a wavelength of 488 nm, FluoZin-3 fluorescence signals emitted at wavelengths longer than 510 nm were collected at 0.1 Hz using the Argus/HiSCA imaging system (Hamamatsu Photonics, https://www.hamamatsu.com/jp/en.html) driven by Imagework Bench v6.0 (INDEC Medical Systems, http://www.indecmedical.com/). Changes in fluorescence intensity were calculated as Zn^2+^_i_ (F/F_0_) by the normalization at time zero. For each analysis, fluorescence signals were averaged over the whole-cell area. To quantitatively measure changes in Zn^2+^ levels, responses from 50 cells within a single coverslip were averaged, and this protocol was replicated to change the coverslip. To measure changes in intracellular Ca^2+^ ([Ca^2+^]_i_), HEK cells, which were loaded with 10 μM Fura2-AM (DOJINDO) in SBS for 30 min at room temperature, were superfused with SBS for 10 min to remove residual Fura2-AM in the recording chamber. Fura2 fluorescence signals, elicited upon excitation at wavelengths of 340 nm and 380 nm, were recorded at 0.1 Hz. Changes in the fluorescence intensity ratio were calculated as Ca^2+^_i_ (F_340_/F_380_) and analyzed in the similar manner to [Zn^2+^]_i_.

### Modeling

Molecular modeling was performed using UCSF Chimera (https://www.cgl.ucsf.edu/chimera/) v1.16 and v1.17 (30) and the modeling software MODELLER (https://salilab.org/modeller/) v10.3 and v10.4 (45, 46), or SWISS-MODEL (https://swissmodel.expasy.org/) (47) to employ the 3D structural data of JT010-bound hTRPA1 (PDB ID: 6PQO) and the predicted 3D structural data of hTRPA1 (AF-O75762-F1-model_v4.pdb) and gTRPA1 (AF-W8VTH6-F1-model_v4.pdb) in AlphaFold DB (31, 32). For predicting MIA sites for Zn^2+^ in TRPA1s, we used the MIB2 (http://combio.life.nctu.edu.tw/MIB2/) (27, 28, 29) to input the 6PQO-derived structural monomer data (residues: 447–1079 AAs in hTRPA1) and AlphaFold-predicted modeling data (a full-length of 1119 AAs and 1126 AAs in hTRPA1 and gTRPA1, respectively). Each TRPA1 AA residue was assigned a binding score, with a subset presented in Table S1. A higher binding score for a residue indicated a greater likelihood of it being a metal-binding site. In Table S2, docking scores of AAs against Zn^2+^ were summarized in WT-hA1 and WT-gA1 for further prediction. To find additional AA residues which potentially bind to Zn^2+^, we also utilized Metal Geometry in UCSF Chimera, a tool for analyzing metal ion coordination geometries. For the input, the docking data predicted by MIB2 (WT-hA1; 983H and 987E for IZD1 and 979Q and 983H for IZD2 (Table S2), WT-gA1; 984H and 988E for IZD1 and 920H, 921D, and/or 925D for EZDL (Table S2), H919-hA1; H919 and E924 for EZDL) were used (Supporting information).

### Data analysis

Data were primarily analyzed and presented using Origin 9.1 (Lightstone, https://www.lightstone.co.jp/origin/feature/origin91/). Results are represented as individual values and the mean ± SD. To ascertain statistical significance between the two groups, paired and unpaired Student’s *t* tests were conducted *via* Origin 9.1. For comparisons among multiple groups, two-way ANOVA, Tukey-Kramer (Tukey) tests (both in Origin 9.1), and Dunnett's test (KyPlot 5.0, KyensLab Inc, https://www.kyenslab.com/en-us/) were used.

### Reagents

The following drugs were used: ZnSO_4_ (Zn^2+^; Fujifilm Wako Pure Chemical Corp), AITC (Kanto Chemical Co), A-967079 (Focus Biomolecules), HC-030031 (Enzo Life Sciences), and sodium pyrithione (Pyr; Sigma-Aldrich). Each drug was dissolved in the vehicle recommended by the manufacturer.

## Data availability

The data presented in this study are available on request from the corresponding author (kmuraki@dpc.agu.ac.jp).

## Supporting information

This article contains supporting information (17, 27, 28, 29, 31, 32, 45, 47).

## Conflict of interest

The authors declare that they have no conflicts of interest with the contents of this article.
