# Supplementary material for: Critical amino acid residues regulating TRPA1 Zn2+ response: A comparative study across species
Source: J Biol Chem. 2024 Apr 18;300(6):107302. doi: 10.1016/j.jbc.2024.107302 (PMC11134551; doi:10.1016/j.jbc.2024.107302)
Supplement: Supporting information [file mmc1.docx]

**Supporting information**

**Critical Amino Acid Residues Regulating TRPA1 Zn^2+^ Response: A Comparative Study Across Species**

Masaki Matsubara^1^, Yukiko Muraki, Hiroka Suzuki, Noriyuki Hatano, Katsuhiko Muraki*^,1^

Laboratory of Cellular Pharmacology, School of Pharmacy, Aichi Gakuin University, 1-100 Kusumoto, Chikusa, Nagoya 464-8650, Japan

^1^These authors equally contributed to this study. *Corresponding author: Katsuhiko Muraki, Ph.D., Laboratory of Cellular Pharmacology, School of Pharmacy, Aichi Gakuin University, 1-100 Kusumoto, Chikusa, Nagoya 464-8650, Japan

E-mail: kmuraki@dpc.agu.ac.jp

***Results and Discussion***

**Protein expression of TRPA1s**

In Figure S1, we evaluated the protein expression levels of wild-type (WT) and mutant TRPA1 variants. For WT TRPA1, western blotting (WB) was performed in HEK cells transfected with WT human TRPA1 (WT-hA1), WT mouse TRPA1 (WT-mA1), and WT chicken TRPA1 (WT-gA1). For this analysis, we employed two distinct antibodies targeting the C-terminal and N-terminal regions of TRPA1 (Fig.S1A and S1B, respectively). In addition, while we utilized two other antibodies (Fig.S1C), these did not detect the expression of WT-mA1 and WT-gA1. We also quantified the protein expression of various hTRPA1 mutants, including IZD1-, IZD2-, EZDL-, and SNP-mutants with the C-terminal (Fig.S1D-S1F) and N-terminal (Fig.S1G) TRPA1 antibodies.


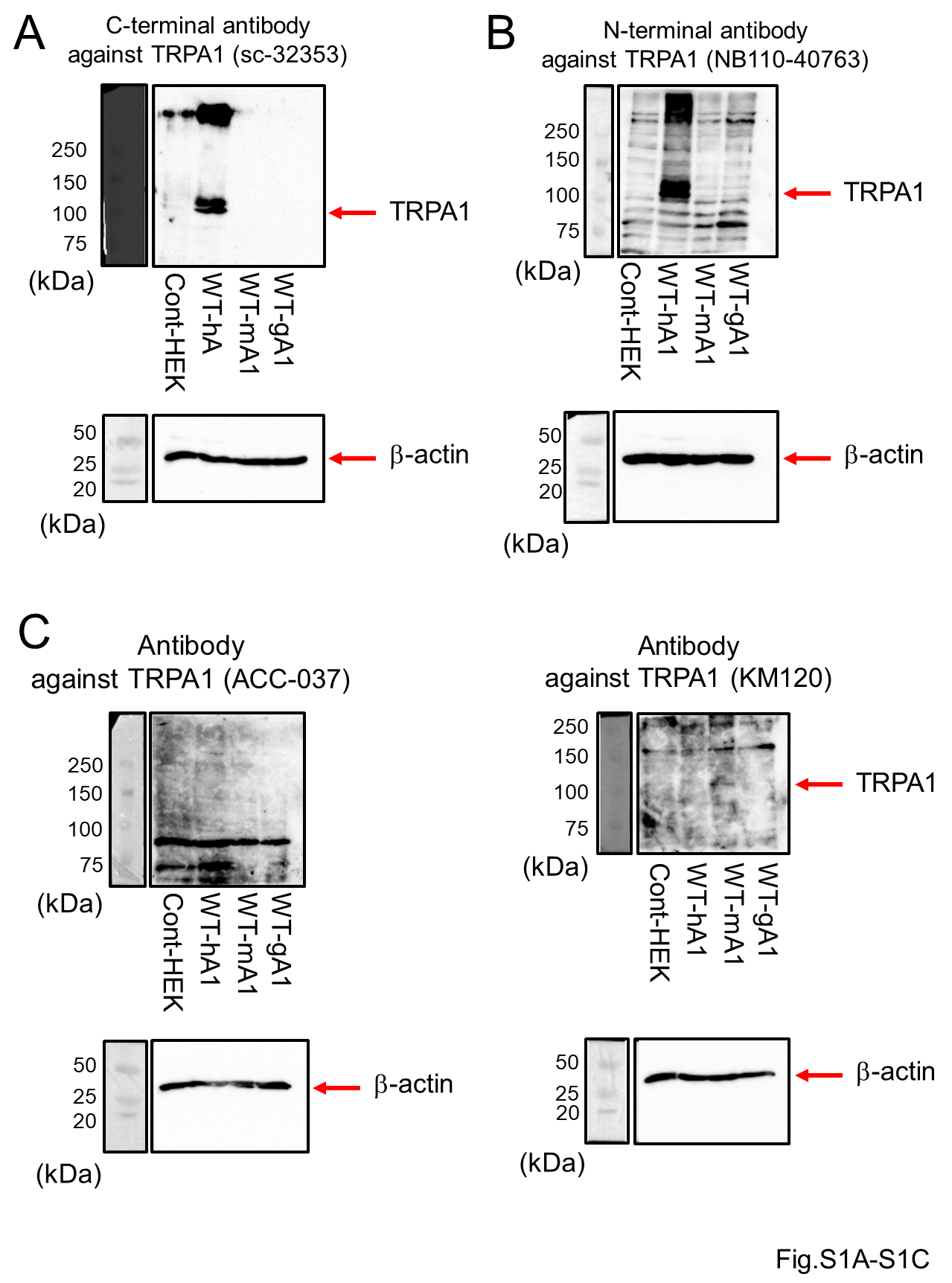


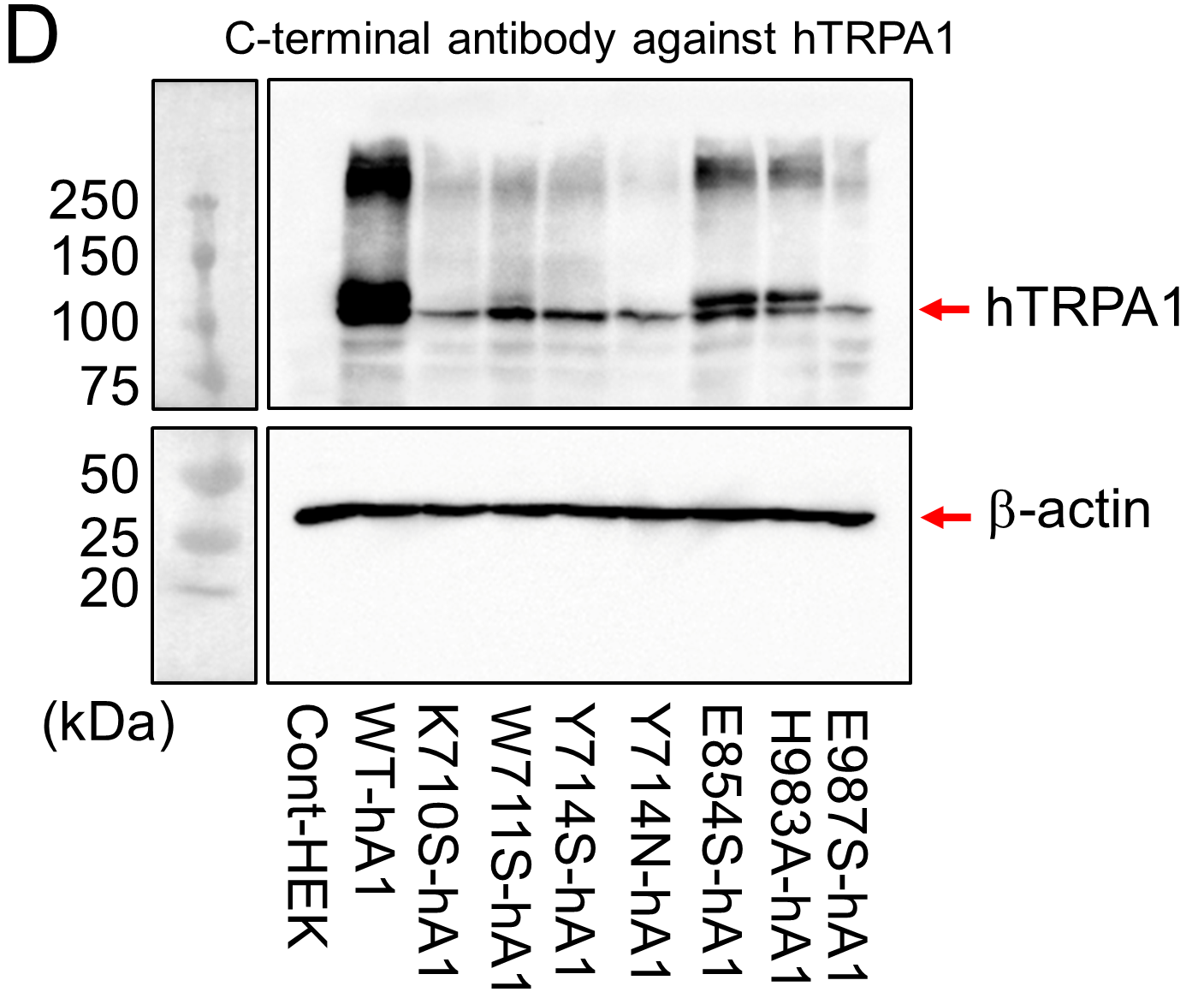

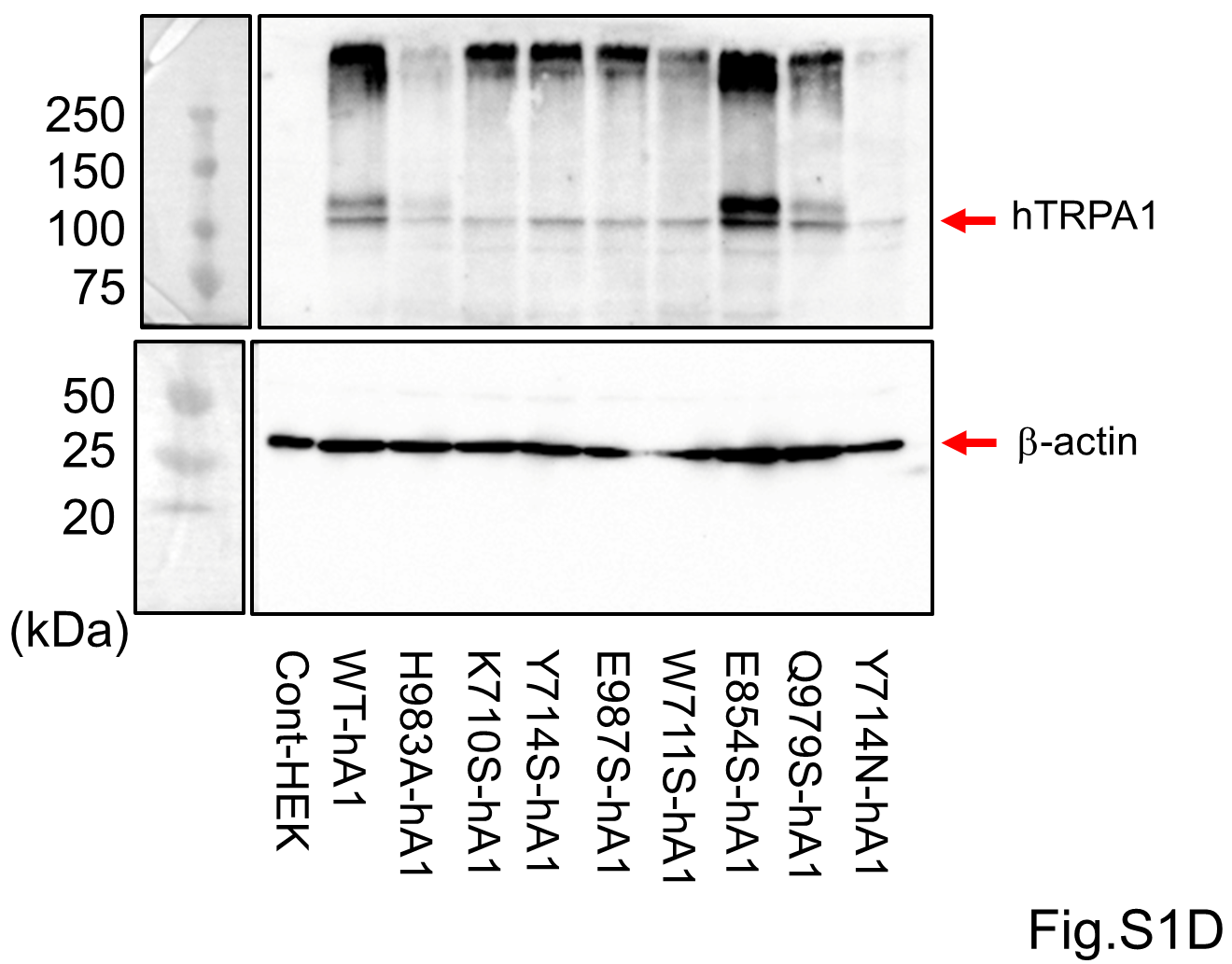

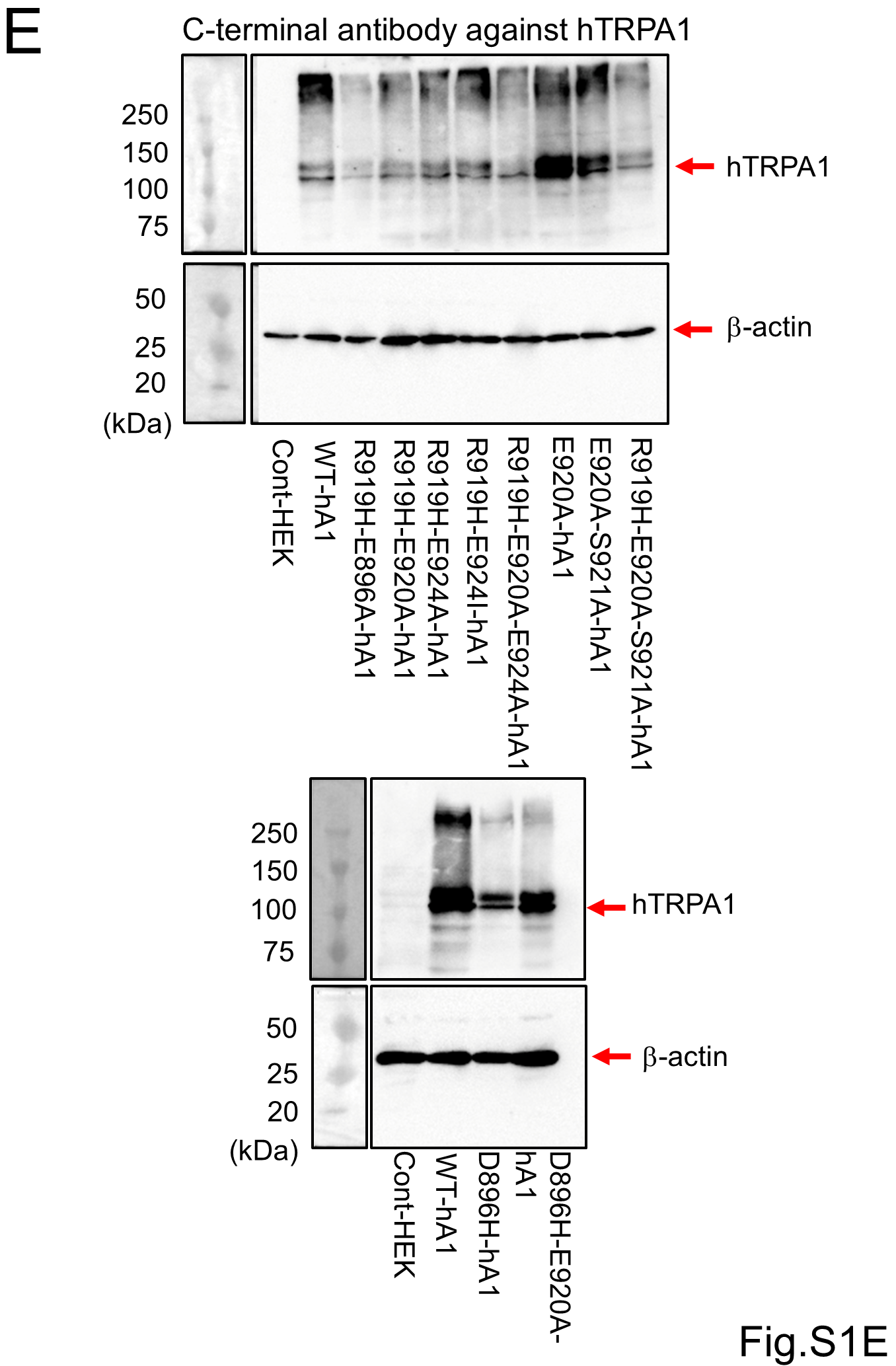

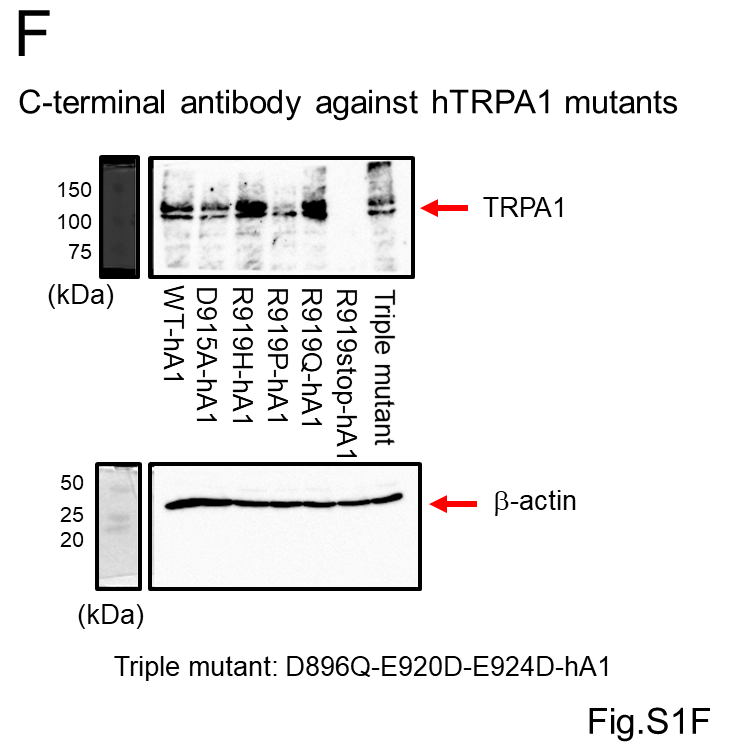


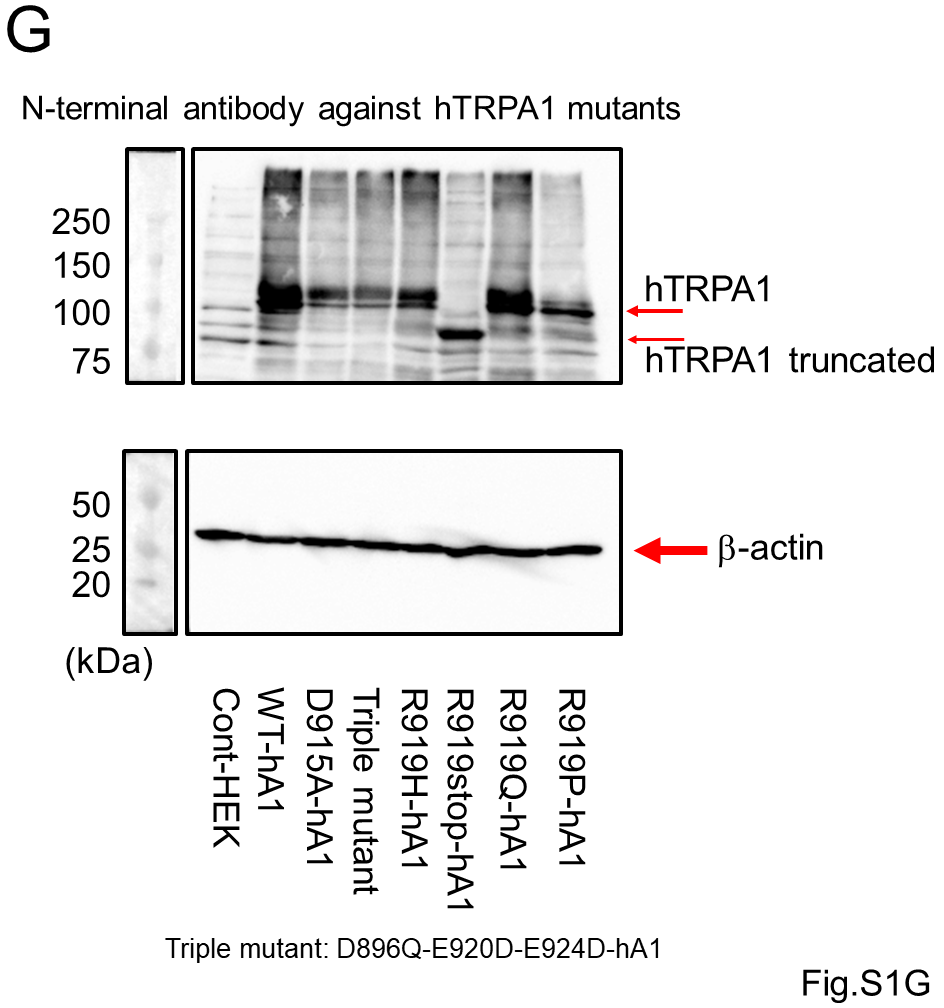


**Figure S1.** Evaluation of protein expression of WT and mutant TRPA1s. For WT TRPA1, WB analysis was performed in HEK cells transfected with WT-hA1, WT-mA1, and WT-gA1. Two different antibodies targeting the C-terminal and N-terminal regions of TRPA1 and other two antibodies commercially available were utilized (**A-C**). The expression levels of β-actin, used as a loading control, were also qualified. Each product No was shown in parenthesis. (**D-G**) The protein expression of various hTRPA1 mutants, including IZD1, IZD2, EZDL, and SNP mutants was assayed (**D** in upper panel: K710S-hA1, W711S-hA1, Y714S-hA1, Y714N-hA1, E854S-hA1, H983A-hA1, E987S-hA1; **D** in lower panel: H983A-hA1, K710S-hA1, Y714S-hA1, E987S-hA1, W711S-hA1, E854S-hA1, Q979S-hA1, Y714N-hA1; **E** in upper panel: R919H-D896A, R919H-E920A, R919H-E924A, R919H-E924I, R919H-E920A-E924A, E920A-hA1, E920A-S921A-hA1, R919H-E920A-S921A-hA1; **E** in lower panel: D896H-hA1, D896H-E920A, **F**: D915A-hA1, R919H-hA1, R919P-hA1, R919Q-hA1, R919stop-hA1, D896Q-E920D-E924D-hA1. The same protein expression analysis was performed using the N-terminal antibody (**G**: D915A-hA1, D896Q-E920D-E924D-hA1, R919H-hA1, R919stop-hA1, R919Q-hA1, R919P-hA1). HEK cells with WT-hA1 and without TRPA1s (Cont-HEK) were used as controls. Expression levels were quantified through at least two independent experiments (**A**-**G**).

**Sequence alignment of human and chicken TRPA1**

The sequence alignment of human and chicken TRPA1 orthologs was performed and secondary structure elements were indicated (Fig.S2). Specifically, putative AAs constituting IZD1, IZD2, and EZDL are highlighted in red characters. As a reference, cysteine at position 1021 (C1021) in hTRPA1 is also shown in underline. Notably, this C1021 is substituted to glutamine (Q1021) in WT-gA1.





**Figure S2.** Sequence alignment of human and chicken TRPA1 orthologs. Green, ankyrin repeat; H, helix; S, transmembrane segment; P, pore helix; TRP helix, TRP-like helix domain; IFH, interfacial helix; PreS1, Pre S1 helix; CC, coiled coil; β, β sheet. Putative AAs composing IZD1, IZD2 and EZDL are shown in red. Please refer to Fig. 4E for a detailed topological model of human and chicken TRPA1.

**Effect of Zn^2+^ on WT-gA1 with and without A-967079 or Pyr**

In Figure S3A, the impact of 5 μM A-967079 and the combination of 5 μM A-967079 with 30 μM Zn^2+^ on WT-gA1 was investigated. If both characteristics, “a non-electrophilic gTRPA1 agonist” and “a potent inhibitor of hTRPA1” refer to A-967079, it is unclear that 30 μM Zn^2+^ slightly activated WT-gA1 in the presence of A-967079 (Fig.S3A). Meanwhile, in Figure S3B-C, the effects of Zn^2+^ applied intracellularly on HEK cells expressing WT-gA1 were tested. Specifically, 1 μM Zn^2+^ was administered to WT-gA1 with and without a Zn^2+^ ionophore Pyr at 3 μM. However, the effect was variable among cells and not statistically significant (Fig.S3B and S3C).

**

**

**Figure S3.** (**A**) The effects of 5 μM A-967079 and the combination of 5 μM A-967079 with 30 μM Zn^2+^ on WT-gA1. Peak current amplitudes at +90 mV were plotted and averaged (7 independent experiments; ^##^P<0.01 by Tukey test). To validate TRPA1 response and expression, 30 μM HC-030031 and AITC were employed. (**B**, **C**) The effects of intracellular Zn^2+^ on HEK cells expressing WT-gA1. Specifically, 1 μM Zn^2+^ was applied to WT-gA1 with and without 3 μM Pyr. TRPA1 channel expression was confirmed by 30 μM AITC. I-V relationships under each experimental condition and time-course changes of peak inward and outward currents at -90 and +90 mV are shown in the upper and lower panel, respectively (**B**). Peak current amplitudes at -90 and +90 mV were plotted and averaged (**C**; 5**-**6 independent experiments, ^##^P<0.01 by paired t-test). The label "ns" indicates no significance.

**Modeling of IZDs**

We employed the metal ion-binding site prediction and modeling server (MIB2, (1-3)) to identify potential Zn^2+^ binding sites in TRPA1s. The structural data for this prediction included the protomer structures based on 6PQO (residues of 447-1079 AAs) and the AlphaFold DB modeling data (AF-O75762-F1-model_v4 for WT-hA1 (a full-length of 1119 AAs) and AF-W8VTH6-F1-model_v4 for WT-gA1 (a full-length of 1126 AAs) (4,5)). Our findings, detailed in Table S1, highlight the predicted Zn^2+^ binding AAs in IZD1, IZD2, and EZDL. In addition, C1021 in WT-hA1, a previously suggested crucial Zn^2+^ binding residue (6), is also included. Each TRPA1 residue was assigned a binding score (Table S1), indicating its potential as a Zn^2+^ binding site; higher scores suggested a stronger binding propensity. Specifically, the AlphaFold DB model for WT-hA1 predicted Zn^2+^ binding AAs in IZD1, with the C1021 score notably higher than that in the 6PQO structural data (Table S1). Table S2 presents a subset of potential Zn^2+^ docking AAs and their MIB2 scores, with relevant AAs in IZDs and EZDL highlighted by double underline. These docking data were used in Metal Geometry in UCSF Chimera to construct Zn^2+^-binding 3D models (Fig.4F and Fig.8A). Table S3 details the coordination of Zn^2+^ with AAs in IZDs and EZDL, predicting the domain formation with specific AA sets in WT-hA1 and WT-gA1: K710, Y714, H983, E987 (IZD1) in WT-hA1; K716, Y720, H984, E988 (IZD1) and H920, D921, D925 (EZDL) in WT-gA1. These reconstructed models were shown in Fig.4F (IZD1) and Fig.8A (EZDL). In contrast, while MIB2 and Metal Geometry predictions were ambiguous regarding Zn^2+^ binding sites in IZD2 for both WT-hA1 and WT-gA1 (Table S2-S3), the 6PQO structure and AlphaFold DB models indicated a preference for Zn^2+^ binding at W711 and Q979 in IZD2 (Table S3). Furthermore, the AlphaFold DB model showed a high binding score for E854 (1.83, Table S1). Based on these insights, we proposed a putative IZD2 (Fig.4E and 4F) comprising specific AA sets in WT-hA1 (W711, E854, H983, Q979) and WT-gA1 (W717, E855, H984, Q980). To validate these predictions, we constructed various mutants targeting IZD1 and IZD2 (Fig.5-6). Meanwhile, the AlphaFold DB model suggested a potential third IZD (IZD3) in WT-hA1, consisting of W711, C1021, and C1025 (Table S3). It is evident that the substitution of C1021 with serine resulted in a reduced Zn^2+^ response (6), and the W711S mutant exhibited no response to either Zn^2+^ or AITC (Fig.5I and 5K). However, our findings emphasize the significance of IZD2 in Zn^2+^ detection in both hTRPA1 and gTRPA1. Notably, the cysteine residues at positions 1021 and 1025, present in hTRPA1, are absent in gTRPA1 (Q1021 and G1025 for gTRPA1, Fig.4E and Fig.S2). Moreover, in gTRPA1, mutants E855S and W717S specifically lost their Zn^2+^ response (Fig.6A).

**Modeling of EZDL**

We employed the MIB2 and Metal Geometry for modeling the EZDL in TRPA1s. In WT-hA1, glutamate residues at positions 920 and 924 exhibited relatively high Zn^2+^ binding scores (1.807 each, Table S1), although MIB2 did not predict their interaction with Zn^2+^ (Table S2). A close-up view of this model is illustrated in Fig.8A. For WT-gA1, MIB2 identified histidine at position 920 (H920) and aspartate residues at positions 921 (D921) and 925 (D925) as potential components of the EZDL (Table S1 and S2). By combining Metal Geometry with MIB2 docking data for WT-gA1 (Table S3), we estimated the likely coordination distances between these AAs and Zn^2+^, forming the EZDL structure, as depicted in Fig.8A. Furthermore, we hypothesized that a mutant hTRPA1 with an R919H substitution (R919H-hA1) might form an EDZL using residues H919, E920, E924, and/or D896. This was supported by high Zn^2+^ binding scores for these AAs in the EZDL (Table S1), as predicted by MIB2 when reconstructing a homology model of the dimer H919-hA1 structure (AA residues from 800 to 1000) with the 6PQO as a template (7,8). Using Metal Geometry with MIB2 docking data for the H919-hA dimer, we further predicted the potential coordination of these AAs with Zn^2+^ (Fig.8B). To test this hypothesis, we introduced mutations at E920, E924, and D896 in H919-hA1 and analyzed their responses to Zn^2+^ (Fig.8C and 8D).

**Table S1.** Prediction of Zn^2+^ binding to AAs in TRPA1s by MIB2

| AA position | AA in WT-hA1 | MIB2 score (6PQO) | MIB2 score (AF-O75762-F1-model_v4) | AA position | AA in WT-gA1 | MIB2 score (AF-W8VTH6-F1-model_v4) |
| --- | --- | --- | --- | --- | --- | --- |
| IZD1,2 |  |  |  | IZD1,2 |  |  |
| 983 | HIS | 4.294 | 3.719 | 984 | HIS | 3.187 |
| IZD1 |  |  |  | IZD1 |  |  |
| 710 | LYS | -0.417 | -0.213 | 716 | LYS | -0.256 |
| 714 | TYR | 0.81 | -0.488 | 720 | TYR | -0.473 |
| 987 | GLU | 4.294 | 3.719 | 988 | GLU | 3.187 |
| IZD2 |  |  |  | IZD2 |  |  |
| 711 | TRP | -0.417 | -0.488 | 717 | TRP | -0.473 |
| 854 | GLU | -0.025 | 1.83 | 855 | GLU | 0.494 |
| 979 | GLN | 1.929 | 1.423 | 980 | GLN | 1.233 |
| EZDL |  |  |  | EZDL |  |  |
| 896 | ASP | -0.129 | -0.488 |  |  |  |
| 919 | ARG | 0.2 | 0.770 | 920 | HIS | 3.075 |
| 920 | GLU | 1.807 | 0.874 | 921 | ASP | 3.075 |
| 924 | GLU | 1.807 | 0.874 | 925 | ASP | 3.075 |
| Zn^2+^ related |  |  |  |  |  |  |
| 1021 | CYS | -0.038 | 2.931 |  |  |  |
| AA position | AA in H919-hA1 | MIB2 score (homology model) |  |  |  |  |
| 896 | ASP | 2.017 |  |  |  |  |
| 919 | HIS | 4.721 |  |  |  |  |
| 920 | GLU | 2.894 |  |  |  |  |
| 924 | GLU | 4.721 |  |  |  |  |

double underline: over-threshold indicated by MIB2; underline: close to the threshold (between 1.8 and 1.9); IZD1: intracellular Zn^2+^ binding domain1; IZD2: intracellular Zn^2+^ binding domain2; EZDL: extracellular Zn^2+^ binding domain-like; 6PQO: PDB ID; AF-O75762-F1-model_v4 and AF-W8VTH6-F1-model_v4: AlphaFold DB ID

**Table S2.** Zn^2+^ docking scores of AAs in TRPA1s predicted by MIB2

| Rank | AA pair (WT-hA1) | Identifier | Score | Rank | AA pair (WT-gA1) | Identifier | Score |
| --- | --- | --- | --- | --- | --- | --- | --- |
| 1 | 1062H,1063E | 2yx1B2 | 5.488 | 1 | 830H,834E | 4dygA0 | 4.874 |
| 2 | 585H,589H | 2eg4A0 | 4.521 | 2 | 590H,594H,624N | 2fbhA1 | 4.670 |
| 3 | 1061Q,1062H | 3fjuB0 | 4.303 | 3 | 830H,834E | 2qswA1 | 4.487 |
| 4 | 983H,987E | 4kjmB0 | 4.294 | 4 | 492H,496Q | 3luuA0 | 4.444 |
| 5 | 625E,628E | 2zc2B0 | 3.939 | 5 | 85Q,89D | 4e45D0 | 4.414 |
| 6 | 625E,628E | 1sedC0 | 3.783 | 6 | 148H,150E | 5grqB1 | 4.272 |
| 7 | 487H,519H | 1qwyA0 | 3.764 | 7 | 830H,834E | 1yo7A0 | 4.208 |
| 8 | 983H,987E | 5mltA0 | 3.719 | 8 | 830H,834E | 5c22B0 | 4.140 |
| 9 | 983H,987E | 4clvB0 | 3.617 | 9 | 590H,594H | 3psqB4 | 4.076 |
| 10 | 625E,628E | 4zpjA1 | 3.542 | 10 | 478R,482E | 4gqtA1 | 4.000 |
| 11 | 447K,451H | 2x4hB2 | 3.525 | 11 | 1043E,1046K | 2vrzB0 | 3.757 |
| 12 | 983H,987E | 2qswA1 | 3.524 | 12 | 558H,562K | 2gx8C0 | 3.755 |
| 13 | 983H,987E | 1cy5A2 | 3.513 | 13 | 141H,145Q,168E | 3hwpB0 | 3.654 |
| 14 | 545D,547D | 1sw1A1 | 3.495 | 14 | 395Q,396H | 3fjuB0 | 3.642 |
| 15 | 983H,987E | 6ckoC0 | 3.486 | 15 | 282H,319H | 2y5qA0 | 3.559 |
| 16 | 983H,987E | 5g5yA2 | 3.455 | 16 | 590H,594H | 4h4lH0 | 3.537 |
| 17 | 625E,628E | 3i9fA1 | 3.436 | 17 | 590H,630E | 2imzB0 | 3.513 |
| 18 | 983H,987E | 4dygA0 | 3.408 | 18 | 245C,249H | 1i7wA0 | 3.472 |
| 19 | 983H,987E | 4fmnB0 | 3.388 | 19 | 590H,594H | 4a6dA0 | 3.372 |
| 20 | 545D,547D | 5ch8A0 | 3.375 | 20 | 830H,834E | 5fnpA1 | 3.316 |
| 21 | 447K,451H | 3v1fB1 | 3.35 | 21 | 803D,805S | 3mcxA1 | 3.275 |
| 22 | 983H,987E | 2ymkB0 | 3.336 | 22 | 984H,988E | 1yo7A0 | 3.187 |
| 23 | 585H,589H | 1yj0A0 | 3.286 | 23 | 830H,834E | 6ckoC0 | 3.187 |
| 24 | 1061Q,1062H | 5mxzA1 | 3.257 | 24 | 536Q,540D | 4e45D0 | 3.175 |
| 25 | 481H,512H,513N | 5kdjA0 | 3.186 | 25 | 830H,834E | 3m3bA0 | 3.108 |
| 26 | 983H,987E | 1r8qE0 | 3.164 | 26 | 920H,921D,925D | 2haeD0 | 3.075 |
| 27 | 545D,547D | 2c1dG1 | 3.125 | 27 | 1062H,1066K | 5fnpA1 | 2.975 |
| 28 | 481H,512H,513N | 5y1tA0 | 3.1 | 28 | 415D,417E,423H, 424Y | 2yheC1 | 2.972 |
| 29 | 635K,639D,700H | 1vykA0 | 3.097 | 29 | 590H,594H,630E | 2h1nA0 | 2.964 |
| 30 | 625E, 628E | 1zzhB0 | 3.017 | 30 | 984H,988E | 4clvB0 | 2.950 |
| 48 | 983H, 987E | 1uxbA0 | 2.555 | 32 | 920H,925D | 2vesB1 | 2.870 |
| 50 | 983H, 987E | 1ycgB1 | 2.543 | 39 | 920H,925D | 3m4wF0 | 2.705 |
| 52 | 983H, 987E | 1yo7A0 | 2.495 | 46 | 984H,988E | 2ymkB0 | 2.570 |
| 54 | 983H, 987E | 5c22B0 | 2.480 | 50 | 984H,988E | 2qswA1 | 2.508 |
| 63 | 983H, 987E | 3favB0 | 2.222 | 54 | 920H,925D | 2ymkB0 | 2.468 |
| 70 | 983H, 987E | 5gpoA0 | 2.128 | 62 | 984H,988E | 5mltA0 | 2.383 |
| 79 | 983H, 987E | 2ejcA2 | 2.012 | 90 | 920H,925D | 1py2B0 | 2.041 |
| 88 | 979Q, 983H | 3psqB4 | 1.929 | 95 | 984H,988E | 3m4wF0 | 2.015 |
|  |  |  |  | 98 | 920H,925D | 2d0wA1 | 2.005 |
|  |  |  |  | 109 | 920H,925D | 1yo7A0 | 1.941 |

AAs composing IZDs and EZDL are denoted with a double underline. The 6PQO and AF-W8VTH6-F1-model_v4 structural data were utilized for predictions in WT-hA1 and WT-gA1, respectively.

**Table S3.** The coordination distance between AA and Zn^2+^ in IZDs and EZDL predicted by Metal Geometry in UCSF Chimera





**No involvement of non-charged AA in external Zn^2+^ response of gTRPA1**

To examine the involvement of non-charged glutamine at position 897 (Q897) of gTRPA1 (Fig.7A) in the lower response to external Zn^2+^, we investigated the response of gTRPA1 with Q897D mutation (Q897D-gA1) to external Zn^2+^ (Fig.S4A and S4B). Similar to WT-gA1, the application of Zn^2+^ did not induce membrane currents in this mutant. Conversely, the substitution of aspartate at position 896 (D896) of hTRPA1 to glutamine (D896Q-hA1) rendered the channel sensitive to external Zn^2+^ comparable to WT-hA1 (upper and left panel in Fig.S4C and S4D, respectively). Furthermore, even in triple mutants of hTRPA1 including D896Q, in which D896, E920, and E924 of hTRPA1 were mutated to Q896, D920, and D924 (D896Q-E920D-E924D-hA1) homologous to each AA of gTRPA1, external Zn^2+^ effectively induced membrane currents (Fig.S4C and S4D). There results demonstrated that these distinct AAs of TRPA1 between human and chicken (Fig.7A) are not the primary factors responsible for high sensitivity to external Zn^2+^ in hTRPA1.





**Figure S4.** (**A-B**) The non-charged glutamine at position 897 (Q897) in gTRPA1 was mutated to the charged aspartate (Q897D-gA1), homologous to D896 of WT-hA1 (Fig.7A), to test the sensitivity to external Zn^2+^. I-V relationships under each experimental condition and time-course changes of peak inward and outward currents at -90 and +90 mV are shown in the lower and upper panel, respectively (**A**). The peak current amplitudes at -90 and +90 mV were plotted and averaged (**B**; 8-9 independent experiments; ^##^P<0.01 by paired *t-*test). (**C-D**) Substitution of D896 in hTRPA1 with glutamine (D896Q-hA1) to test the sensitivity to external Zn^2+^. I-V relationships under each experimental condition are shown in the upper panel (**C**). The peak current amplitudes at -90 and +90 mV were plotted and averaged in the left panel (**D**; 3**-**4 independent experiments, ^#^P<0.05 and ^##^P<0.01 by Tukey test). Three AAs at the flanking region of the channel pore (D896, E920, and E924) were mutated to Q896, D920, and D924, homologous to Q897, D921, and D925 in gTRPA1, to test the Zn^2+^ response in the mutant (D896Q-E920D-E924D-hA1). I-V relationships under each experimental condition are presented in the lower panel (**C**). The peak current amplitudes at -90 and +90 mV were plotted and averaged in the right panel (**D**; 3-5 independent experiments; ^#^P<0.05 by Tukey test).

Reference

1. Lin, Y. F., Cheng, C. W., Shih, C. S., Hwang, J. K., Yu, C. S., and Lu, C. H. (2016) MIB: Metal Ion-Binding Site Prediction and Docking Server. *J Chem Inf Model* **56**, 2287-2291

2. Lu, C. H., Chen, C. C., Yu, C. S., Liu, Y. Y., Liu, J. J., Wei, S. T., and Lin, Y. F. (2022) MIB2: metal ion-binding site prediction and modeling server. *Bioinformatics* **38**, 4428-4429

3. Lu, C. H., Lin, Y. F., Lin, J. J., and Yu, C. S. (2012) Prediction of metal ion-binding sites in proteins using the fragment transformation method. *PLoS One* **7**, e39252

4. Jumper, J., Evans, R., Pritzel, A., Green, T., Figurnov, M., Ronneberger, O., Tunyasuvunakool, K., Bates, R., Zidek, A., Potapenko, A., Bridgland, A., Meyer, C., Kohl, S. A. A., Ballard, A. J., Cowie, A., Romera-Paredes, B., Nikolov, S., Jain, R., Adler, J., Back, T., Petersen, S., Reiman, D., Clancy, E., Zielinski, M., Steinegger, M., Pacholska, M., Berghammer, T., Bodenstein, S., Silver, D., Vinyals, O., Senior, A. W., Kavukcuoglu, K., Kohli, P., and Hassabis, D. (2021) Highly accurate protein structure prediction with AlphaFold. *Nature* **596**, 583-589

5. Varadi, M., Anyango, S., Deshpande, M., Nair, S., Natassia, C., Yordanova, G., Yuan, D., Stroe, O., Wood, G., Laydon, A., Zidek, A., Green, T., Tunyasuvunakool, K., Petersen, S., Jumper, J., Clancy, E., Green, R., Vora, A., Lutfi, M., Figurnov, M., Cowie, A., Hobbs, N., Kohli, P., Kleywegt, G., Birney, E., Hassabis, D., and Velankar, S. (2022) AlphaFold Protein Structure Database: massively expanding the structural coverage of protein-sequence space with high-accuracy models. *Nucleic Acids Res* **50**, D439-D444

6. Hu, H., Bandell, M., Petrus, M. J., Zhu, M. X., and Patapoutian, A. (2009) Zinc activates damage-sensing TRPA1 ion channels. *Nat Chem Biol* **5**, 183-190

7. Webb, B., and Sali, A. (2017) Protein Structure Modeling with MODELLER. *Methods Mol Biol* **1654**, 39-54

8. Waterhouse, A., Bertoni, M., Bienert, S., Studer, G., Tauriello, G., Gumienny, R., Heer, F. T., de Beer, T. A. P., Rempfer, C., Bordoli, L., Lepore, R., and Schwede, T. (2018) SWISS-MODEL: homology modelling of protein structures and complexes. *Nucleic Acids Res* **46**, W296-W303
